# Dracorhodin targeting CMPK2 attenuates inflammation: A novel approach to sepsis therapy

**DOI:** 10.1002/ctm2.1449

**Published:** 2023-10-20

**Authors:** Wendan Zhang, Honghong Jiang, Pengli Huang, Gaosong Wu, Qun Wang, Xin Luan, Hongwei Zhang, Dianping Yu, Hongru Wang, Dong Lu, Haonan Wang, Huazhang An, Sanhong Liu, Weidong Zhang

**Affiliations:** ^1^ Shanghai Frontiers Science Center of TCM Chemical Biology Institute of Interdisciplinary Integrative Medicine Research Shanghai University of Traditional Chinese Medicine Shanghai P. R. China; ^2^ Faculty of Pediatrics National Engineering Laboratory for Birth Defects Prevention and Control of Key Technology Beijing Key Laboratory of Pediatric Organ Failure the Chinese PLA General Hospital Beijing P. R. China; ^3^ Shandong Provincial Key Laboratory for Rheumatic Disease and Translational Medicine the First Affiliated Hospital of Shandong First Medical University & Shandong Provincial Qianfoshan Hospital Jinan Shandong P. R. China; ^4^ Department of Phytochemistry School of Pharmacy Second Military Medical University Shanghai P. R. China; ^5^ The Research Center for Traditional Chinese Medicine Shanghai Institute of Infectious Diseases and Biosecurity Shanghai University of Traditional Chinese Medicine Shanghai P. R. China; ^6^ Institute of Medicinal Plant Development Chinese Academy of Medical Sciences and Peking Union Medical College Beijing P. R. China

**Keywords:** CMPK2, dracohodin, mass spectrum, sepsis

## Abstract

**Background:**

Despite all modern advances in medicine, an effective drug for treating sepsis has yet to be found. The discovery of CMPK2 spurred hopes for the treatment of sepsis. However, CMPK2‐untapped target inhibitors are still an enormous obstacle that has hindered the CMPK2‐centric treatment of sepsis.

**Methods:**

Here, we found that the *CMPK2* gene is highly expressed in the whole blood of sepsis patients by RNA‐Seq. First, recombinant CMPK2 was purified by a eukaryotic expression purification system, and the activity of recombinant CMPK2 was detected by the ADP‐GLO assay. Second, we developed an affinity MS strategy combined with quantitative lysine reactivity profiling to discover CMPK2 ligands from the active ingredients of Chinese herbs. In addition, the dissociation constant *K_d_* of the ligand and the target protein CMPK2 was further detected by microscale thermophoresis technology. Third, we used this strategy to identify a naturally sourced small molecule, dracorhodin (DP). Using mass spectrometry‐based quantitative lysine reactivity profiling combined with a series of mutant tests, the results show that K265 acts as a bright hotspot of DP inhibition of CMPK2. Fourth, immune‐histochemical staining, ELISAs, RT‐qPCR, flow cytometry and immunoblotting were used to illustrate the potential function and related mechanism of DP in regulating sepsis injury.

**Results:**

Our results suggest that DP exerts powerful anti‐inflammatory effects by regulating the NLRP3 inflammasome via the lipopolysaccharide (LPS)‐induced CMPK2 pathway. Strikingly, DP significantly attenuated LPS‐induced sepsis in a mouse model, but its effect was weakened in mice with myeloid‐specific *Cmpk2* ablation.

**Conclusion:**

We provide a new framework that provides more valuable information for new therapeutic approaches to sepsis, including the establishment of screening strategies and the development of target drugs to provide a theoretical basis for ultimately improving clinical outcomes for sepsis patients. Collectively, these findings reveal that DP is a promising CMPK2 inhibitor for the treatment of sepsis.

## Graphical abstract

2. A two‐step chemical tracking strategy was developed for the first time to identify and evaluate the inhibitor dracorhodin (DP) of CMPK2.

3. DP, as the first CMPK2 inhibitor, regulates the NLRP3 inflammasome by lipopolysaccharide (LPS)‐induced CMPK2 pathway in vivo and in vitro.

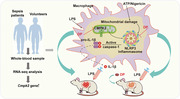

## 1 INTRODUCTION

Medical progress has dramatically increased life expectancy, yet inflammasome‐associated diseases driven by a range of uncontrollable factors remain among the most challenging health issues.^1^ Inflammation is initiated when the body's immune system senses pathogen‐associated or damage‐associated molecular patterns, which underlie many chronic and degenerative diseases that seriously reduce the quality and duration of life.^2^ These diseases include periodic autoinflammatory syndromes, Alzheimer's disease, Parkinson's disease, diabetes mellitus type 2, atherosclerosis, ulcerative colitis and cancers.^3^, ^4^, ^5^, ^6^

The inflammasome is mainly mediated by pattern recognition receptors. These include members of the NOD‐like receptor (NLR) family such as NLRP1 and NLRC4. Other non‐NLR receptors, such as AIM2 and IFI16, are equally important.^7^ Among these receptors, NLRP3 has received a wide variety of interest. Similar to other inflammasomes, the NLRP3 inflammasome can turn on a protective mode for the immune system to resist pathogen invasion and alleviate tissue damage. However, it can also play a detrimental role when inappropriately activated.^4^ Once activated, the sensor NLRP3 oligomerizes, exposing its pyrin domain and recruiting adaptor apoptosis associated speck‐like protein (ASC), which binds with the effector molecule pro‐caspase‐1 to form the NLRP3 inflammasome.^2^, ^8^ Caspase‐1 further cleaves the biologically inactive precursors of cytokines as well as various other substrates, including interleukin‐1β (IL‐1β), IL‐18 and gasdermin D,^9^, ^10^ which induce pyroptotic cell death via pore formation.^11^, ^12^ NLRP3 is a vital promoter and sensor of multiple complex human diseases and serves as a critical core component of the NLRP3 inflammasome; it accelerates population aging and reduces lifespan. Thus, mediating and regulating NLRP3 inflammasome activity is a potential therapeutic approach for inflammatory diseases with unmet clinical needs.

The currently available clinical treatment for NLRP3‐related diseases targets IL‐1β and NLRP3.^7^ As a key trigger of inflammatory diseases, IL‐1β is a central coordinator of immune responses to various classes of pathogens.^13^ IL‐1β agents have demonstrated significant efficacy in the clinical treatment of cryopyrin‐related auto‐inflammatory syndrome, and subsequent clinical trials of other NLRP3‐associated diseases have also been carried out.^14^, ^15^ However, there are concerns about the current treatment modalities. IL‐1β production is regulated by multiple pathways.^16^, ^17^ Therefore, NLRP3 inflammasome inhibitors may be a better option for treating NLRP3‐driven disease than agents targeting IL‐1β. Recently, it has been reported that some active ligands exhibit an underlying inhibitory effect on the activation of the NLRP3 inflammasome.^18^, ^19^ Specifically, MCC950 has demonstrated potent inhibitory effects and benefits in numerous mouse models of NLRP3‐related disease.^20^, ^21^ The short half‐life and high toxicity of MCC950 have limited its future clinical translation in rheumatoid arthritis phase II clinical trials. Therefore, there is an urgent need to develop highly effective small‐molecule ligands and vital targets for blocking inflammasome assembly.

Cytosine/uridine monophosphate aminase 2 (CMPK2) belongs to a family of novel nucleoside monophosphate (NMP) kinases located in mitochondria.^22^ CMPK2 is a neoteric monophosphate kinase residing in mitochondria that is involved in the salvage synthetic pathway of mitochondrial DNA and phosphorylates dUMP, dCMP, CMP and UMP.^23^ Over the last decade, significant progress has been made in delineating the important roles of CMPK2 in the innate immune system.^23^, ^24^, ^25^ Both bacterial PAMPs (lipopolysaccharide [LPS]) and viral PAMPs (viral dsDNA or dsRNA) can trigger the induction of CMPK2. CMPK2 participates in activating the NLRP3 inflammasome and assisting viperin in anti‐viral activity in response to external and endogenous stimuli. It has been demonstrated that CMPK2 plays an important role in the progression of inflammation‐related disorders due to its intimate relationship with the innate immune system.^2^, ^26^ It has been established that NLRP3 is dependent on the catalytic activity of CMPK2, suggesting that CMPK2 is a potential promising therapeutic target to control NLRP3 inflammasome‐associated diseases.^2^ To confirm that CMPK2 is a druggable target in inflammatory disorders, the discovery of CMPK2 inhibitors is a top priority.

In our recent work, we found that in the whole blood of sepsis patients, the *CMPK2* gene is significantly overexpressed compared with that in healthy human volunteers. Thus, we developed an affinity MS strategy combined with quantitative lysine reactivity profiling to identify new ligands of CMPK2 from a collection of natural product extracts to relieve inflammasome‐associated diseases. We used this strategy to identify a naturally sourced small molecule, DP, which inhibits NLRP3 inflammasome activation via CMPK2 and, thus, is a potential treatment for inflammasome‐associated diseases such as sepsis.

## 2 MATERIALS AND METHODS

### 2.1 Compound library preparation

The experiment was performed with 2700 compounds, which were provided by the Second Military Medical University. All compounds were dissolved in DMSO at the same concentration. The 2700 compounds were randomly assigned to six cocktails (cocktail‐1 to cocktail‐6). Each cocktail contained 500 compounds with an internal standard substance (positive control), and every cocktail was pooled with different *m/z* sub‐fractions. The compounds between different cocktails did not overlap. All cocktails were stored at −20°C.

### 2.2 Reagents and animals

These sections of reagents and animals are described in Supporting Information.

### 2.3 Sample processing procedure

The purified kinase CMPK2 was incubated with the six cocktails for 30 min at 37°C in a suitable buffer solution. In the compound cocktail, each compound concentration was 1 μM, and the concentration of the kinase CMPK2 was set at 5 μM. Each of the above incubation solutions was added to a 0.5 mL 10 kDa ultrafiltration tube (Amicon μltra‐0.5 10K Centrifugal Filter Devices, Millipore), and four experimental replicates were conducted for each CMPK2 and BSA pair. The 10 kDa ultrafiltration tube was centrifuged with the incubation solution at 13 000 × *g* for 20 min at 4°C. Centrifugation was stopped when the solution volume was approximately 100 μL, and then the mixture was washed five times with cold ammonium acetate (150 mM, pH 7.5). For affinity selection, 100 μL of the mixture was transferred after centrifugation and rinsed into a new eppendorf tube. Next, 300 μL of pure methanol was used to extract the compounds that had been bound to CMPK2 twice, and the mixture was centrifuged after extraction. The protein precipitate was discarded and the liquid was concentrated with a N_2_ stream. The residue was re‐dissolved in 50% methanol before injection. BSA samples were processed in a manner parallel to that of CMPK2 samples.

### 2.4 UHPLC‐Q‐TOF‐MS analysis

Sample measurement was detected by using an UHPLC instrument (Shimadzu) combined with a Triple TOF 5600^+^ MS (SCIEX). Chromatographic separation was conducted with an Acquity UPLC BEH C18 column (1.7 μm, 2.1 × 100 mm, Waters) at a temperature maintained at 40°C and a flow rate of 0.3 mL/min, with mobile phases of water–0.1% formic acid (A) and acetonitrile (B). The linear gradient elution profile was as follows: 0−2 min, 5% B; 2−12 min, 5−30% B; 12−25 min, 30−90% B; 25−30 min, 90% B; and re‐equilibration for 5 min. Subsequently, the gradient was recovered to the initial conditions at the final 1 min. Whether it was a test sample or a quality control (QC) sample, each injection volume was 5 μL. Other experimental parameters were set by referring to previously published articles.^27^

### 2.5 Ligand characterization based on protein affinity determined by UHPLC‐Q‐TOF‐MS

Briefly, the chromatographic peaks of each bound ligand were extracted using PeakView with identification criteria of precise mass measurement deviation and retention time tolerance of 5 ppm and 0.1 min, respectively. The MS response of each ligand was represented with the integrated peak area extracted by the corresponding extract ion chromatogram. The effective hits were selected on the basis of fold change (FC) value ≥ 2, *p* value < .05 and cyclic voltammetry (CV) ≤ 30% and were identified as candidate ligands of CMPK2. By extracting the *m/z* of individual standards from the acquired MS/MS spectrum, the decimal point of RT was used to distinguish the ambiguous compound peaks in the cocktails.

### 2.6 Dimethylation labelling of the CMPK2 complex and liquid chromatography‐tandem MS

These sections are described in Supporting Information.

### 2.7 Inhibitory effect of DP on CMPK2

The extent of DP intervention on lysine residues of CMPK2 conformational changes was visualized and the correlation between this effect and the inhibitory potency of the inhibitor was assessed. The degree of intervention of labelling time (5, 10 and 15 min) on CMPK2 lysine reactivity was determined with 1 mM DP, which completely inhibited CMPK2 activity. Finally, after the concentration of the active small molecule was fixed, the concentration of the target protein CMPK2 was tested for lysine labelling reactivity according to a gradient of 0.025, 0.05 and 0.1 mg/mL. The range of concentrations used to investigate the effect of small‐molecule ligands on CMPK2 activity was 0.1–150 μM. Each data point contains three biological replicates.

### 2.8 Optimization of the chromatographic conditions

This section is described in Supporting Information.

### 2.9 Method validation

To ensure that the instrument was in good condition and that the samples did not deteriorate during the entire experiment, the overall experimental process was strictly controlled. To evaluate the stability of the instrument system, the principal component analysis (PCA) score plot of the QCs exhibited relatively tight clustering during the entire analysis system. The results showed that the UHPLC‐Q‐TOF‐MS instrument was stable during sample analysis (Supporting Information Figure S1A,B). The mass spectrometry (MS) raw data of biological samples were processed and analysed by PCA (Supporting Information Figure S1C,D), which illustrated that the samples were stable over 120 h. Furthermore, the RSD values of selected ions are shown in Supporting Information Table S1. We observed that the RSD values of the ligands were less than 20%. The above results confirmed the reliability of the MS data.

### 2.10 Culture and stimulation of bone marrow‐derived macrophages

This section is described in Supporting Information.

### 2.11 Injection sequence and method stability assessment

Through preliminary experiments, five small molecules (Supporting Information Table S1) with high responses were found. These small molecules fully met the screening criteria of *p* < .05, FC ≥ 2 and CV ≤ 30%. For the extracted ion chromatographic peaks of five compounds (424.7000, 171.1700, 426.7174, 304.2500 and 458.5440) in the CMPK2 and BSA samples, the CV value was calculated based on the peak area. The five compounds were mixed to prepare QC samples according to a previously described protocol.^27^ The five compounds that were representative chromatographic peaks with a wide range of chemical polarities and *m/z* values were mixed to prepare QC samples according to a previously described protocol. Six experimental samples were combined with one QC sample. Additionally, the stability of the experimental system was determined by comparing the changes in peak areas of the primary analytes after storage at 4°C for different times (0, 12, 24, 48, 72 and 120 h), and the differences were expressed as the RSD of the peak areas. Furthermore, the QC data were processed using PCA to evaluate the stability of the analytical method throughout the whole analysis process.

### 2.12 Cell counting kit 8 assay and ELISA

These sections are described in Supporting Information.

### 2.13 Expression and purification of recombinant CMPK2 kinase and related mutants

**FIGURE 1:**
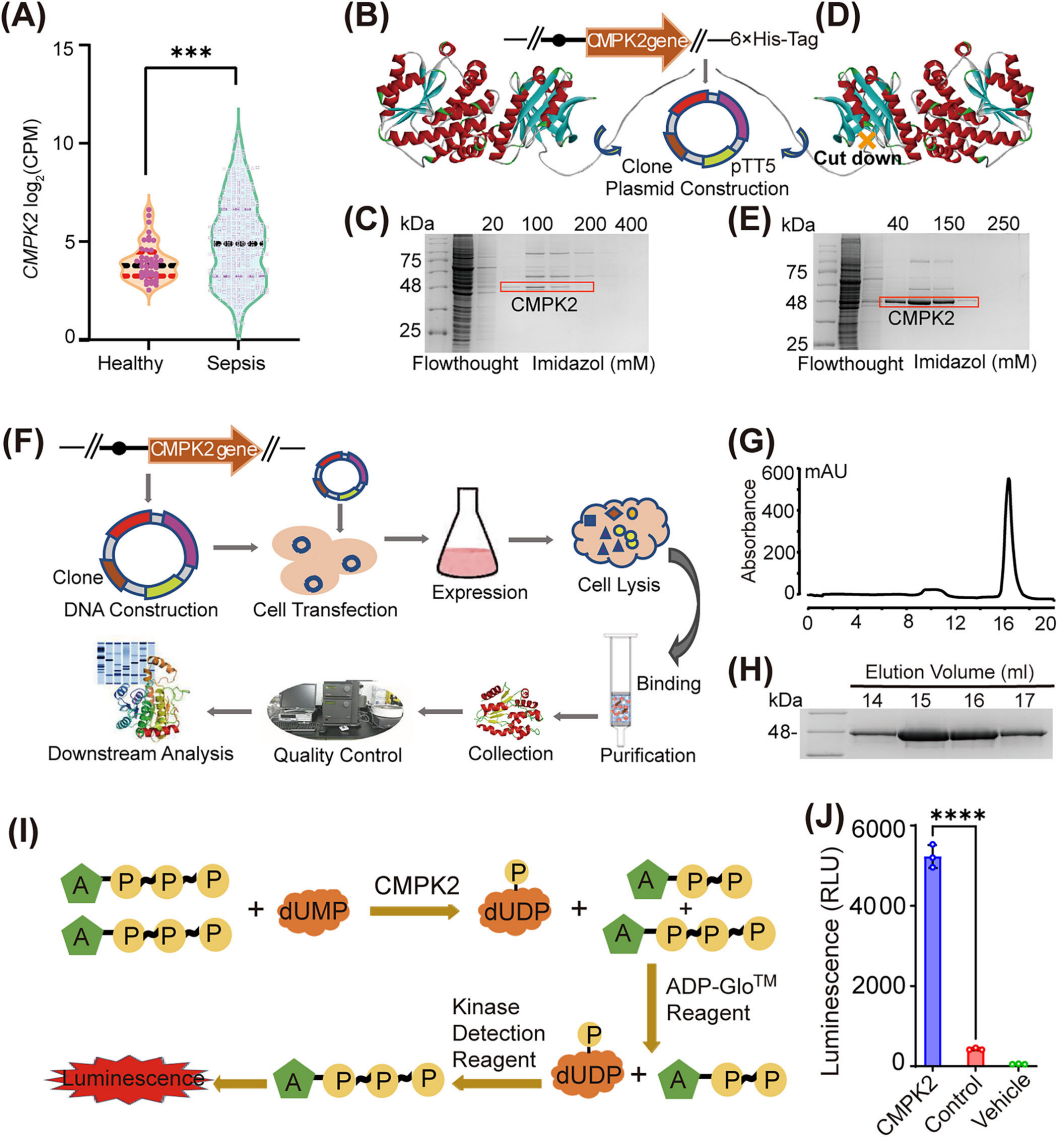
Expression and purification of recombinant CMPK2 kinase. (A) *CMPK2* gene levels in the whole blood of sepsis patients compared with those in healthy human volunteers. (B and D) Construction of the CMPK2 kinase plasmid (B: containing the leading peptide; D: removing the leading peptide). (C and E) Trace CMPK2 from the Ni–NTA purification of SDS–PAGE gel. (F) A schematic diagram of the protocol for the expression and purification of recombinant CMPK2 kinase. (G) Representative gel‐filtration chromatography of recombinant CMPK2 kinase. (H) The peak fractions were visualized by SDS–PAGE and Coomassie staining. (I) Principle of the activity detection of recombinant CMPK2 kinase. (J) Activity assessment of recombinant CMPK2 kinase (n = 3). Statistics were analysed using an unpaired Student's *t* test: *p < .05; **p < .01; ***p < .001. NS, no significance. For (J), data are represented as mean ± SD.

Human CMPK2 and K265, K282 and K320 mutated CMPK2 cDNA were cloned and inserted into the pTT5 vector, and the constructed plasmid was amplified by the DH5α strain. The C‐terminus of the cDNA was fused with a 6× His tag, and the *N*‐terminus was truncated by 22 amino acids (Figure 1B–F and Supporting Information Figure S5A,B). When the free‐style 293‐F cells reached the proper density, approximately 1 mg of expression plasmid was premixed with 3 mg polyethylenimine in UltraFectin medium and incubated prior to transfection. This mixture was then added to the appropriate amount of fresh cell culture medium, incubated for 30 min and collected after 48 h.

For the purification of CMPK2 and related mutants, the cell pellet was lysed under sonication at 200 W in kinase buffer. After ultracentrifugation, the reserved supernatants were successively added to Ni–NTA agarose and combined three times and the flowthrough was collected. Then, CMPK2 and related mutants were eluted with buffers containing different concentrations of imidazole, and 10% SDS–PAGE electrophoresis was conducted to test the protein gel staining (Figure 1H and Supporting Information Figure S5C). After combining with the imidazole eluate containing the target protein, the eluates were concentrated. This sample was injected on a Superdex 200 10/300 GL column equilibrated with kinase buffer for further purification. Then, the samples were stained with 10% SDS–PAGE for protein gels. The elution volumes containing CMPK2 and related mutants were combined, and the concentrations were detected by Nanodrop. Samples were aliquoted into 100 μL tubes and stored at −80°C.

### 2.14 CMPK2 kinase activity assay

CMPK2 activity was measured by the ADP‐GLO kinase assay according to the guidelines specified in the manufacturer's instructions. In the activity assay, the amount of ADP formed in the CMPK2 reaction was equal to the ATP consumption and proportional to the increase in luminescence generated in the luciferase reaction, which is the experimental principle shown in Figure 1I. The light strength correlated to the amount of ADP generated in the kinase assay indicates that it has kinase activity.

### 2.15 Microscale thermophoresis (MST)

This section is described in Supporting Information.

### 2.16 The effect of DP on cellular CMPK2 activity

This section is described in Supporting Information.

### 2.17 Lysine microenvironment changes based on changes in labelling efficiency

CMPK2 was first reported by Anna Karlsson in 2008. The literature has reported its localization in mitochondria.^22^ To date, the CMPK2 crystal structure has not been reported. To perform the lysine microenvironment docking analysis, the structure of CMPK2 was predicted based on AlphaFold.^28^, ^29^ Using AutoDockTools (version 1.5.6), significant differences in labelling efficiency between the CMPK2‐DMSO and CMPK2‐DP states were highlighted and preserved for each *K* site, the remaining protein structure remained rigid, and the DP was retained after reversal. The docking parameters were set by using AutoDock Vina version 1.1.2, num mode was set to 20, energy range was set to 5, exhaustiveness was set to 100 and the grid box size was set *xyz* to (68, 70 and 74). To improve the simulated accuracy of the small‐molecule interaction region, the docking conformations should be consistent with the trend of labelling efficiency as much as possible. The binding poses of the target protein and the specific ligand were presented by PyMol.

### 2.18 ASC oligomerisation assay and immunoblot analysis

These sections are described in the supplementary materials.

### 2.19 SiRNA‐mediated gene silencing and dsDNA‐mediated gene overexpression in bone marrow‐derived macrophages (BMDMs)

These sections are described in Supporting Information.

### 2.20 Mitochondrial membrane potential analysis

The treated bone marrow‐derived macrophages (BMDMs) and JC‐1 working solution were incubated in a cell incubator for 30 min. At the end of the warm incubation, the cells were washed three times with PBS, and images were captured with a Harmony 4.8 (PerkinElmer). Meanwhile, the stained BMDMs were quantified by flow cytometry.

### 2.21 Measurement of mitochondrial ROS

For mitochondrial ROS (mtROS) measurement, according to the instruction guidelines, a diluted 5 μM MitoSOX working solution was incubated with treated BMDMs in a cell incubator for 10 min according to the instructional guidelines. The BMDMs were gently washed with warm buffer to remove the working solution. The images were captured using Harmony 4.8 (PerkinElmer). Meanwhile, the stained BMDMs were quantified by flow cytometry.

### 2.22 LPS‐induced septic shock model

This section is described in Supporting Information.

### 2.23 Chemometric data and statistical analysis

These sections are described in Supporting Information.

## 3 RESULTS

### 3.1 *The CMPK2* gene is highly expressed in the whole blood of sepsis patients

To determine the primary function of the target protein CMPK2 in patients with sepsis, we collected clinical data of whole‐blood RNA‐seq from 348 patients with sepsis from four emergency departments and one intensive care unit, while matching 44 healthy volunteers as clinical controls. Gene expression signatures that predicted subsequent severity were identified by metrological statistical analysis. In addition, clinicians can identify groups of patients most at risk on the basis of their initial clinical presentation and guide the appropriate use of antibiotics. Therefore, we described the hematologic immune profile of early/pre‐septic patients to identify features reflecting disease severity, organ dysfunction, mortality and specific endotypes and mechanisms. Notably, by whole‐blood RNA‐seq, we found that the *CMPK2* gene was dramatically upregulated in sepsis patients compared to healthy volunteers (Figure 1A). Overall, the clinical data showed that higher levels of *CMPK2* in sepsis patients were associated with triggering inflammation and excessive immune reactions.

### 3.2 Preparation and enzyme activity detection of recombinant CMPK2 kinase

CMPK2 is a NMP kinase capable of specifically phosphorylating dUMP, dCMP, CMP and UMP, which is mainly positioned on mitochondria, and its catalytic activity promotes the synthesis of mitochondrial DNA.^2^, ^22^ CMPK2 is a mitochondrial protein with a putative mitochondrial targeting signal (Figure 1B). However, during the optimization of our expression system, we found that the expression and purification of CMPK2 were greatly limited by the existence of mitochondrial targeting signals (Figure 1C).^22^ Therefore, under the premise of preserving the basic domain of the CMPK2 kinase, the mitochondrial targeting signal is removed, which greatly increases the expression of CMPK2 (Figure 1D,E). The mitochondrial targeting signal fragment of the CMPK2 protein is the leading peptide (1–21 amino acids). The molecular weight of the leading peptide is approximately 2 kDa. Recombinant CMPK2 kinase was obtained via a eukaryotic expression and purification system (Figure 1F). The concentrated target protein was injected into a Superdex 200 10/300 GL column for further purification (Figure 1G). Elution volumes 14, 15, 16 and 17 containing CMPK2 were combined, and the concentration was determined. Samples were aliquoted into 100 μL tubes and stored at −80°C (Figure 1H). The kinase activity of this recombinant CMPK2 was detected by ADP‐GLO, and the principle of activity detection is shown in Figure 1I. Comparing the luminescence of the control (no CMPK2) group, vehicle group and CMPK2 group, we found that the luminescence intensity of the CMPK2 group roughly exceeded that of the other two groups (Figure 1J). The fluorescence response intensity of different groups was measured and indicated that CMPK2 maintained kinase activity.

### 3.3 Ultrafiltration‐affinity mass spectrometry profiling to identify small‐molecule inhibitors of CMPK2

**FIGURE 2:**
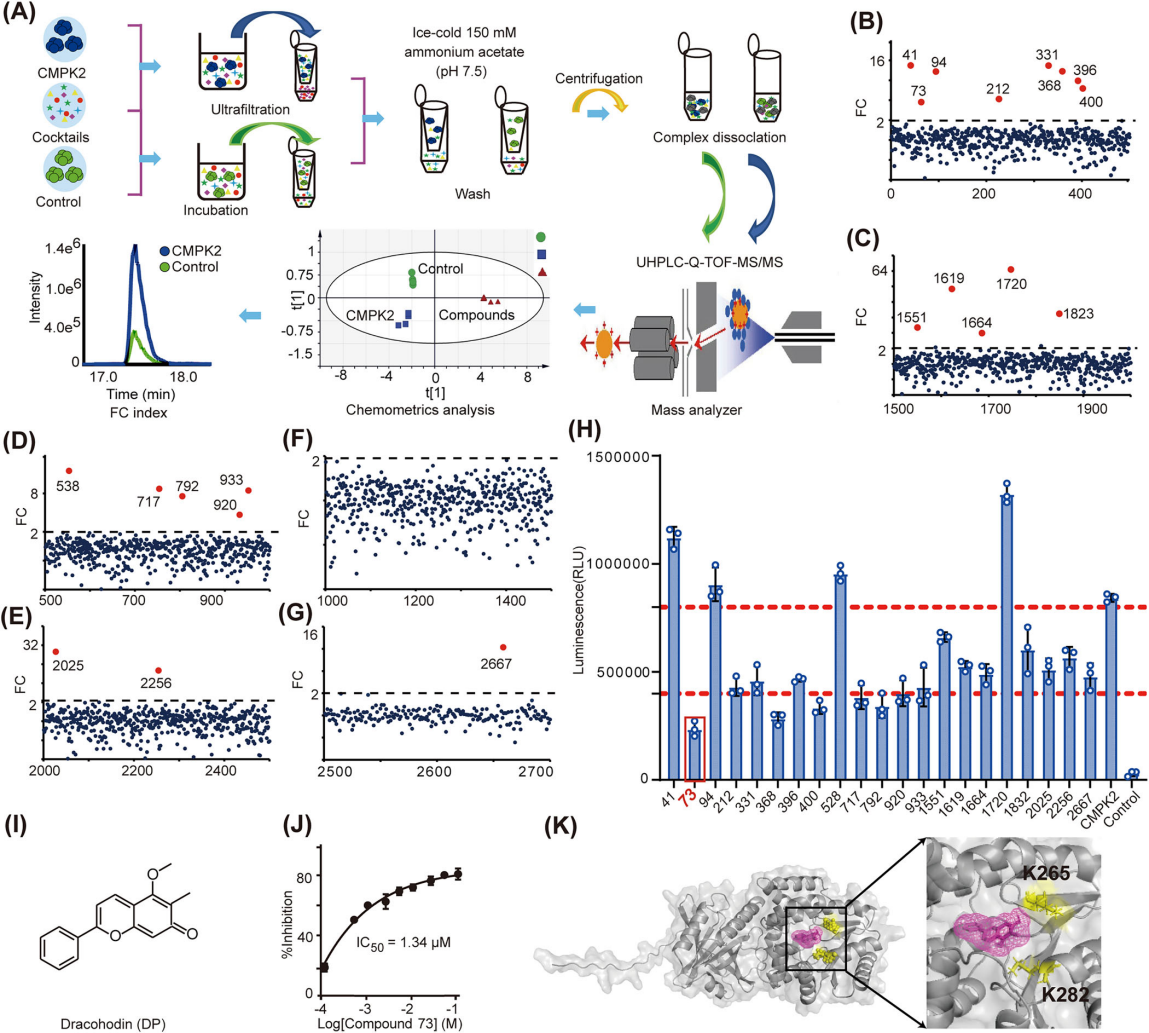
Identification of a competitive CMPK2 inhibitor. (A) The experimental workflow of affinity MS screening for CMPK2 ligands. (B–G) Affinity MS screening of a 2700‐member compound library split into six cocktails against CMPK2. Initial hits (mean FC ≥ 2 and *p* < .05) are indicated by red dots, while grey dots represent negative hits. (H) Hit validation with the ADP‐GLO assay on the 21 putative ligands (n = 3). (I) DP structure. (J) Inhibition of CMPK2 by DP. IC_50_ measurements are represented as the mean and SD from experimental triplicates. (K) The DP (purple) in the CMPK2 complex interferes with the proximal microenvironment of Lys265 and Lys282 (yellow). For (H, J), data are represented as mean ± SD.

We sought to establish a relatively single and stable protein screening system to identify direct small‐molecule ligands of CMPK2 using ultrafiltration‐affinity mass spectrometry.^30^ CMPK2 was incubated with a cocktail. The bound ligand was dissociated from the protein complex using suitable organic reagents and injected into the LC–MS system to characterize the ligand profile.^31^ Another non‐related protein, BSA (to prevent specific adsorption on the membrane), was processed in the same manner as a negative control (Figure 2A). Next, we employed affinity LC–MS to screen a collection of 2700 small molecules against CMPK2. This library was first divided into six cocktails of 500‐ or 200‐mix to represent the complexity of compound pools commonly observed in affinity MS experiments (Figure 2B–G). In addition, PCA was performed on QC samples eluted by an LC–MS gradient to illustrate the good condition of the experimental samples and the instrument system (Supporting Information Figure S1 and Table S1).

The experimental samples were injected into a UHPLC‐Q‐TOF‐MS system. First, total ion chromatograms (TIC) reflected the general overview of all samples (Supporting Information Figure S2A). The results of the PCA score plot showed that the CMPK2 group, BSA group and control group exhibited three distinct clustering trends (Supporting Information Figure S2B), indicating the specific binding of the given compounds to the target protein. Among the groups, each sample with a CV more than 30% was considered an abnormal sample and was not considered in the subsequent statistical analysis. In addition, the *p* value of each sample between the groups was calculated by MetaboAnalyst 5.0, and the significance of differences was determined. In the ultrafiltration‐affinity mass spectrometry profiling, we calculated FC, which is defined as the ratio of the MS response between the compound detected in the CMPK2 and BSA incubation system (Supporting Information Figure S2C,D). The FC parameter allowed us to compare the affinity of different compounds that bound to the CMPK2 target while excluding compounds that did not specifically interact with CMPK2. There are no reported inhibitors of CMPK2 kinase. Therefore, we first detected the affinity between CMPK2 kinase and its specific substrate dUMP, and the experimental results showed that the FC value was 1.9 (Supporting Information Figure S3A). To select small‐molecule ligands with strong affinity, we determined the screening criteria to be FC ≥ 2. Affinity MS analysis generated a set of raw data, and the compounds that met the pre‐judgment criteria (FC ≥ 2, *p* < .05) were selected as compound hits (Supporting Information Figure S2). Hits identified from the 500‐ or 200‐mix cocktails are illustrated in Supporting Information Figure S2B–G. Screening six cocktails resulted in a total of 21 initial hits with a mean FC ranging from 3.0 to 64.41. For the 21 CMPK2 ligands hit in our screening, the effect of these ligands on CMPK2 activity was detected at a concentration of 10 μM, and by comparing the luminescence intensity of each ligand, the results visually clarified that four compounds, 73, 368, 400 and 792, exhibited sharp CMPK2 inhibition of more than 50% (Figure 2H). Detailed information about these four ligands is shown in Supporting Information Table S2.

We conducted kinase activity assays to evaluate the half‐inhibitory concentrations (IC_50_) of the compounds with respect to CMPK2, and we found that hit compound 73 (Dracohodin, DP) had an IC_50_ = 1.34 μM (Figure 2I,J). Based on the screening criteria and kinase activity detection, DP was ultimately determined to be an active small‐molecule inhibitor of CMPK2 (Supporting Information Figure S3B). The structure of CMPK2 was predicted based on AlphaFold,^28^, ^29^, ^32^, ^33^ and molecular docking of compounds 73, 368, 400 and 792 with CMPK2 showed that compound 73 exhibited the lowest binding energy, −8.2 kcal/mol, and the amino acids in CMPK2 that interacted with 73 were Lys282 and Lys265 (Figure 2K and Supporting Information Table S3). As demonstrated by affinity mass spectrometry, DP interfered with the binding between dUMP and CMPK2 (Supporting Information Figure S3C). Based on the subsequent quantitative lysine reactivity profiling of CMPK2 in the presence or absence of DP, DP may inhibit the catalytic activity of CMPK2 by competing directly with dUMP.

### 3.4 A pivotal conformational intervention site, Lys265, in the CMPK2 complex

**FIGURE 3:**
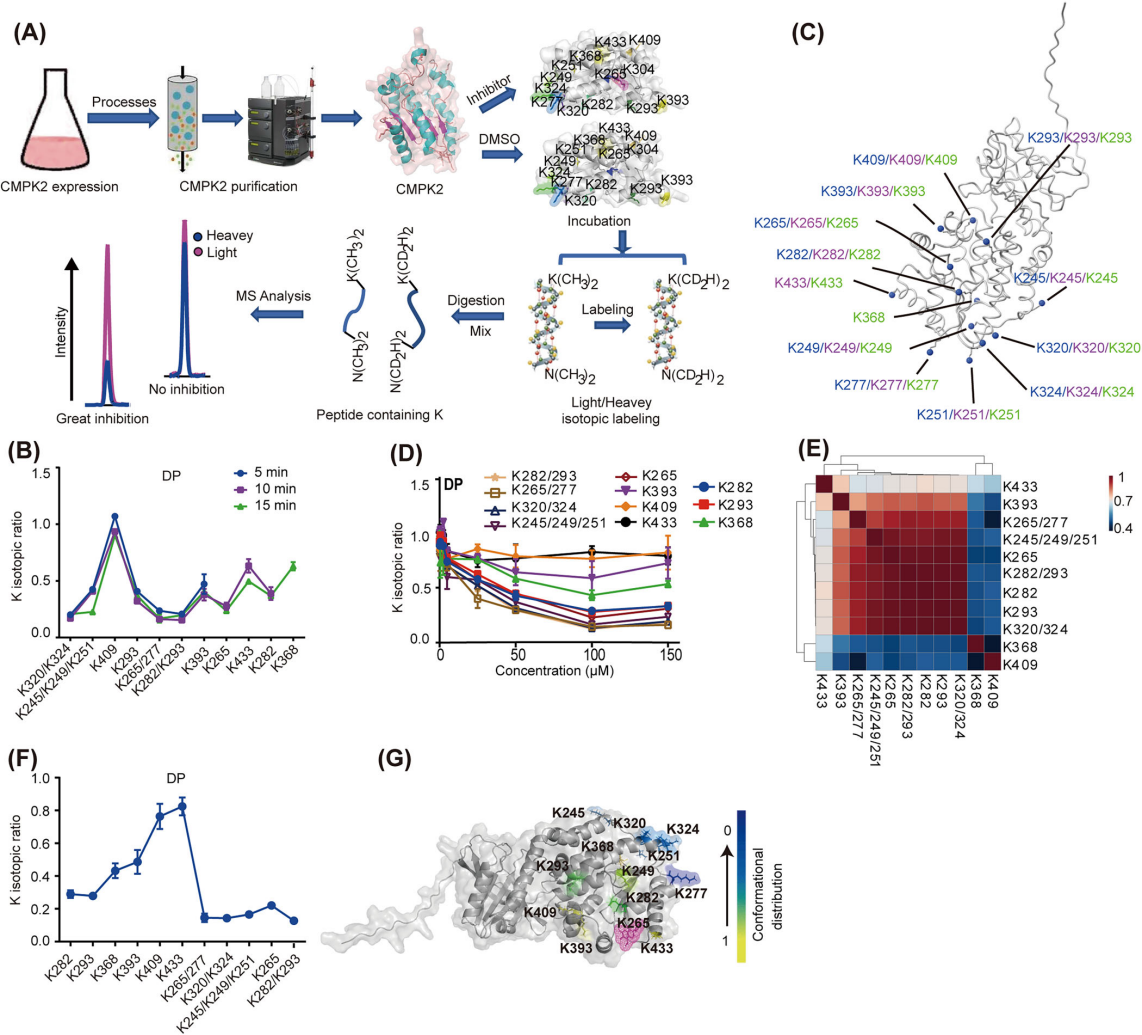
Probing conformational hotspots for the recognition and intervention of CMPK2‐DP complexes. (A) Workflow of the quantitative lysine reactivity profiling strategy. (B) Time‐dependent lysine isotope ratios in CMPK2 (20 μM) incubated with 1 mM DP. (C) The dimethyl labelling times were 5, 10 and 15 min, and the conformational distribution of the quantified lysine sites. (D) Concentration‐dependent curves of lysine isotope ratios as determined by quantitative lysine reactivity profiling. (E) Evaluating the conformational inhibitory potency of DP against the CMPK2 kinase. (F) The lysine isotope ratio values of 13 CMPK2 lysine residues. (G) The conformational distribution of the quantified lysine sites. Each data point was an average value of six replicate experiments, which contained two biological replicates and each with three replicate MS runs (B, D and F). For (B, D and F), data are represented as mean ± SD.

In the dimethyl‐labelled reaction system, the conformational inhibition of the active inhibitor is mediated through direct interaction or induced conformational change to interfere with the reactivity of lysine residues positioned in the regulatory region of the protein.^34^, ^35^ Stable isotope labelling reactions were performed in samples of CMPK2 in the active state. A schematic diagram of the quantitative lysine reactivity profiling strategy is shown in Figure 3A. In this system, when the labelling time was 5 or 10 min, the local regions of the remaining *K* sites were not significantly regulated by the inhibitors (Figure 3B,C). In contrast, a total of 13 lysine residues were comprehensively monitored, and the maximum labelling efficiency of these 13 lysine active sites was reached at the optimum labelling time of 15 min (Figure 3B,C). The conformational distribution of the quantified lysine sites is shown in Supporting Information Figure S4A–C. Among these residues, the isotope ratios of K265, K282 and K320 reached the lowest labelling efficiency when the concentration was 100 μM (Figure 3D). In addition, through an analysis performed on DP binding, we found a positive correlation between the isotopic ratio changes induced by different concentrations of DP and the activity of DP inhibitory protein (Figure 3E). In short, the positive correlation analysis results described above further confirm that the labelling strategy can quantify the conformational inhibition efficiency of kinase inhibitors.

To quantify the degree of conformational effect on lysine residues, we further evaluated the relationship between small‐molecule inhibitor concentration and isotope‐labelling efficiency. As the concentration of DP increased from 0.1 to 150 μM, the isotope ratios of the remaining lysine active sites decreased correspondingly. Among these residues, the isotope ratios of K265, K282 and K320 reached the lowest point when the concentration was 100 μM. The isotope ratio barely changed as the DP concentration increased to 150 μM (Figure 3D). Therefore, 100 μM DP is the optimal labelling concentration for quantifying lysine labelling reactions. When DP was incubated with 0.025 mg/mL CMPK2, the TICs of the sample clearly showed that the target protein was lost, possibly due to the loss of protein during the sample desalination process (Supporting Information Figure S4D). When the concentration of the incubated target protein CMPK2 was adjusted to 0.05 mg/mL, not all effective CMPK2 digestive peptides containing *K* were identified (Supporting Information Figure S4E). When the incubation concentration of CMPK2 was adjusted to 0.1 mg/mL, the results showed that DP was effective against CMPK2‐digested *K*‐containing peptides (Supporting Information Figure S4F and Table S4). Therefore, in terms of data reliability, the minimum allowable concentration of CMPK2 was 0.1 mg/mL.

Based on the quantitative proteomics analysis of changes in the isotopic ratio of lysine residues, in the co‐incubation labelling system of DP and CMPK2, the isotope‐labelling ratios of residues K393, K433 and K409 were approximately 1, while the labelling ratios of K245, K249, K282, K320, K324, K293, K265, K251, K277 and K368 were significantly lower than 0.5 (Figure 3F). The decrease in the isotopic labelling ratio indicated that the corresponding microenvironment of the proximal lysine was disturbed when the active DP molecule bound to the CMPK2 pocket. Therefore, regions proximal to K265, K277, K282, K309, K324, K293 and K320 in CMPK2 were involved in the dynamic inhibition of DP, in which K320, K265 and K282 were the main regions involved in mediating DP inhibition efficiency (Figure 3G).

**FIGURE 4:**
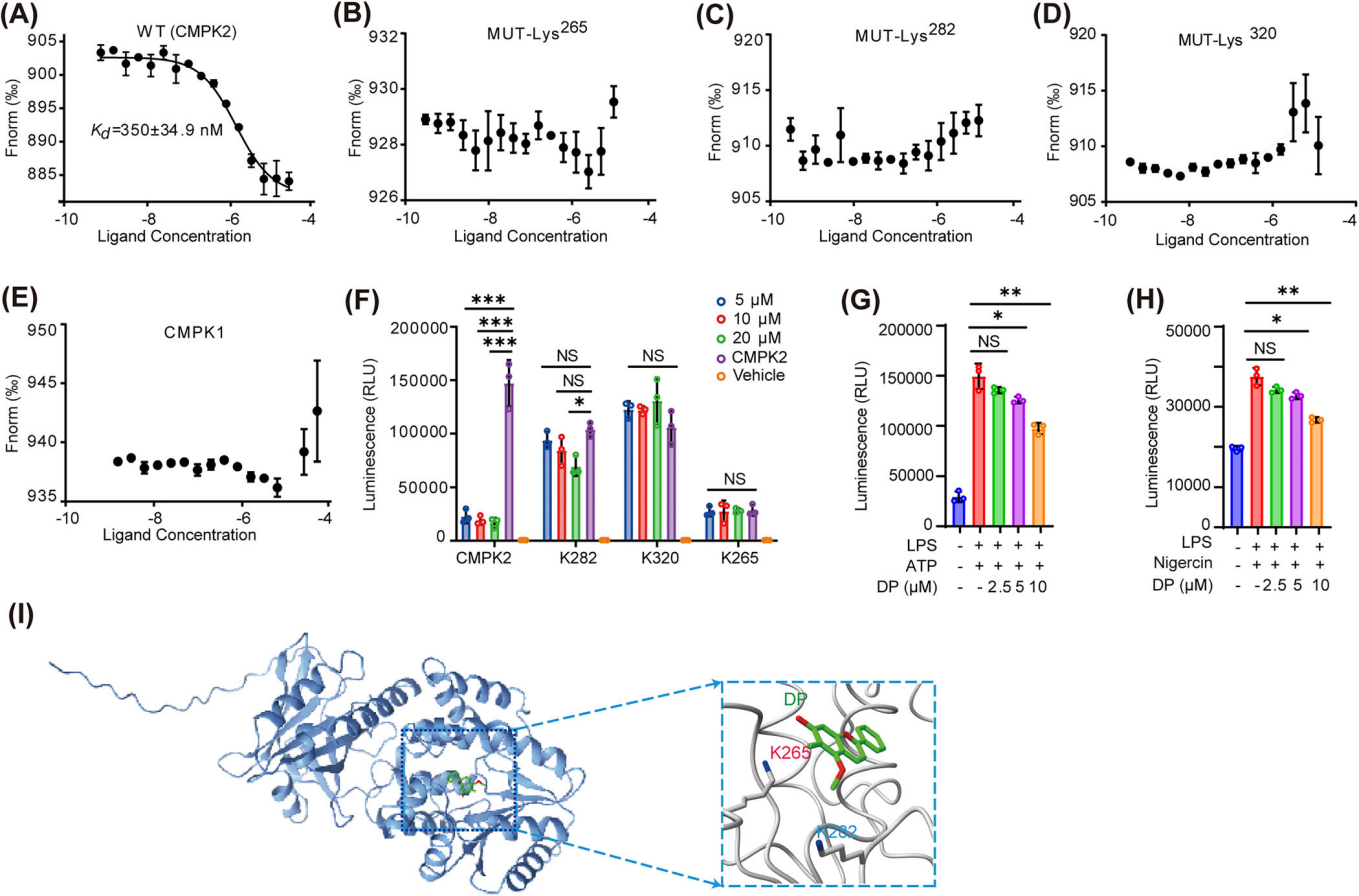
K265 is a novel conformational hotspot in the CMPK2‐DP interaction. (A) MST assay showing the affinity between DP and purified CMPK2 protein. (B) MST assay showing the affinity between DP and purified CMPK2^Lys265^ protein. (C) MST assay showing the affinity between DP and purified CMPK2^Lys282^ protein. (D) MST assay showing the affinity between DP and purified CMPK2^Lys320^ protein. (E) MST assay showing the affinity between DP and purified CMPK1 protein. (F) Activity evaluation of recombinant CMPK2 kinase and related mutants (n = 3). (G and H) The effect of DP on cellular CMPK2 activity (n = 3). (I) Lysine proximal microenvironment of Lys265 in CMPK2 kinase. Each data point contains three biological replicates (A–H). For (A–H), data are represented as mean ± SD. *p < .05; **p < .01; ***p < .001.

To confirm the direct interaction of DP with CMPK2, we performed microscale thermophoresis (MST) analysis to characterize the thermodynamics of DP binding with CMPK2. The results showed that DP bound to CMPK2 with a *K_d_* of 350 ± 34.9 nM (Figure 4A), demonstrating a strong interaction between DP and CMPK2. Lys265, which is located in the P‐loop of human CMPK2, is highly conserved among NMP kinases. As the counterpart of Lys265 in human CMPK2, Lys19 in human thymidylate kinase plays a vital role in the process of phosphoryl group transfer between ATP and TMP to yield ADP and TDP.^36^ To verify this hypothesis, we mutated Lys265, Lys282 and Lys320. The mutant was expressed, and the encoded protein was purified (Supporting Information Figure S5). Using MST technology, the *K_d_* values of the mutants and DP were detected, and it was found that there was no binding between DP and CMPK2 after the three lysine residues were mutated (Figure 4B–D).

Both human CMPK1 and CMPK2 kinases can specifically catalyse UMP, CMP, DUMP and dCMP, converting them into another form of existence, called the diphosphate form, playing dominant roles in regulating precursors of nucleic acid synthesis.^22^, ^37^, ^38^, ^39^ These studies indicated that there is a certain similarity between the substrate‐binding pockets of CMPK1 and CMPK2. Therefore, to exclude multi‐functional targets for DP intervention, MST technology was adopted to detect the binding of DP and CMPK1 before performing a mechanistic study of DP action in vivo and in vitro (Figure 4E). The experimental results showed that there was no bonding between DP and CMPK1, indicating that there was specific binding between DP and CMPK2. Taken together, these observations suggest that CMPK2 is a crucial regulator for exploring the DP mechanism of action. Subsequently, we detected the activity of the mutant protein using ADP‐GLO and found that the kinase activity was significantly lost when K265 was mutated (Figure 4F). Then, we examined the effect of DP on cellular CMPK2 activity. There was a tendency to inhibit the efficiency of enzyme activity at DP concentrations of 2.5 and 5 μM, although this tendency was not very significant, but this inhibition efficiency was enhanced significantly when the DP concentration was increased to 10 μM (Figure 4G,H). Thus, it seems reasonable that the replacement of Lys265 with an Ala residue impaired the catalytic activity of CMPK2 (Figure 4I).

### 3.5 DP inhibits NLRP3 inflammasome activation in macrophages

**FIGURE 5:**
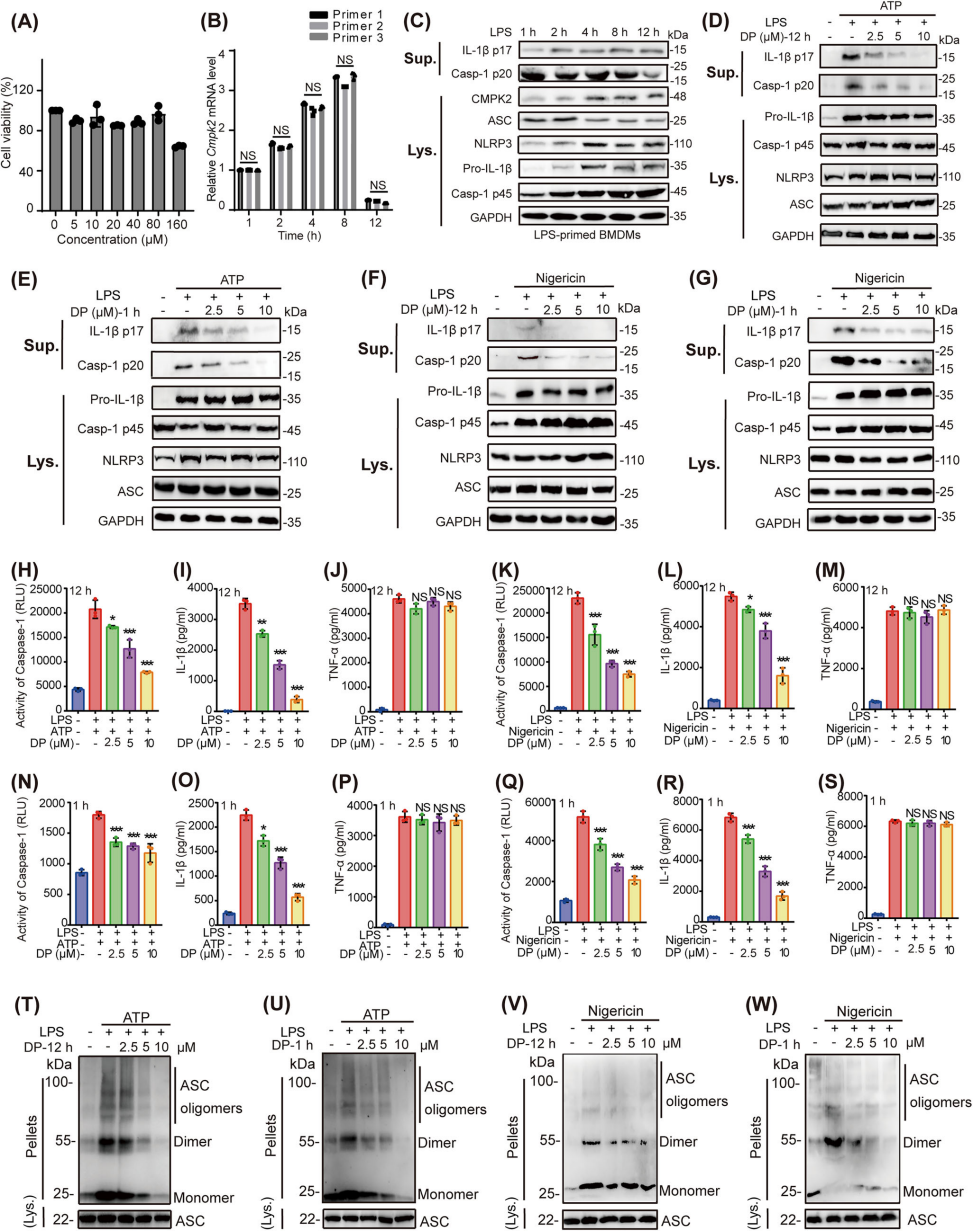
DP inhibited NLRP3 inflammasome activation. (A) Cytotoxicity of DP in BMDMs. (B) Time‐course analysis of changes in *Cmpk2* mRNA levels in BMDMs after LPS initiation of the NLRP3 inflammasome. (C) Time‐course analysis of CMPK2 and NLRP3 inflammasome‐associated subunit accumulation in BMDMs after LPS priming. (D–G) BMDMs were treated with various doses (above lanes) of DP (2.5, 5 and 10 μM) for 12 h, and then LPS‐primed BMDMs were stimulated with 4 mM ATP or 10 μM Nigericin, or LPS‐primed BMDMs were treated with various doses of DP for 1 h and then stimulated with 4 mM ATP or 10 μM Nigericin. Western blot analysis of cleaved p17 and activated p20 in culture supernatants (Sup.) and pro‐IL‐1β, pro‐caspase‐1 (p45), NLRP3 and ASC in lysates (Lys.) of BMDMs. (H–S) BMDMs were treated with various doses of DP (2.5, 5 and 10 μM) for 12 h, and then LPS‐primed BMDMs were stimulated with 4 mM ATP or 10 μM Nigericin. Activity of caspase‐1 (H and K), IL‐1β secretion (I and L) and TNF‐α production (J and M) in Sup. of BMDMs. LPS‐primed BMDMs were treated with different concentrations (2.5, 5 and 10 μM) of DP for 1 h, followed by stimulation with 4 mM ATP or 10 μM Nigericin for 45 min. Activity of caspase‐1 (N and Q), IL‐1β secretion (O and R) and TNF‐α production (P and S) in the Sup. of BMDMs (n = 3). (T and V) Immunoblot analysis of ASC oligomerization in lysates of BMDMs treated with various doses (upper lanes) of DP for 12 h, and then LPS‐primed BMDMs were stimulated with 4 mM ATP or 10 μM Nigericin, input or Sup. (U and W) Immunoblot analysis of ASC oligomerization in lysates of LPS‐primed BMDMs treated with various doses (upper lanes) of DP for 1 h and then stimulated with ATP or Nigericin, input or Sup. Statistics were analysed using an unpaired Student's *t* test: *p < .05; **p < .01; ***p < .001. NS, no significance. For (H–S), data are represented as mean ± SD.

Before investigating how DP affects inflammasome activation, we studied the cytotoxicity of DP in BMDMs, which were treated with different concentrations of DP and then analysed by CCK‐8 assay. We found no significant change in cell viability with various DP concentrations (5–80 μM) (Figure 5A). Notably, the mRNA expression level of CMPK2 increased significantly after 4−8 h of LPS stimulation (Figure 5B). Additionally, we detected changes in the protein levels of the target protein CMPK2 as well as NLRP3 inflammasome‐associated subunits (Figure 5C), and the experimental results demonstrated that the protein expression levels of CMPK2 and NLRP3 gradually increased with the extension of LPS stimulation time, while the expression of mature IL‐1β (p17) and caspase‐1 (p20) decreased sharply and abruptly after 4 h (Figure 5C, Supporting Information Figure S6A).

To confirm the effect of DP on inflammasome activation, canonical NLRP3 inflammasome activators (ATP and Nigericin) were used to activate inflammation. We first investigated the effect of DP on p20 activation and p17 release, as well as changes in the protein levels of NLRP3 inflammasome‐associated subunits. BMDMs were treated with DP for 12 h and stimulated with LPS and subsequently activated with ATP or Nigericin (Figure 5D,F, Supporting Information Figure S6B,C,F and G), or BMDMs were stimulated with LPS and treated with DP and subsequently activated with ATP or Nigericin (Figure 5E,G, Supporting Information Figure S6D,E,H,I). The corresponding statistical graphs of the blots are in Supporting Information Figure S6A–I. The results indicated that DP significantly inhibited p20 cleavage and p17 maturation in the supernatant of BMDMs. In addition, BMDMs were given different DP treatments: one was treated with DP overnight for 12 h, and the other was treated with DP for 1 h after LPS priming of the NLRP3 inflammasome, followed by inflammasome activation. Then, ELISA kits were used to detect whether DP inhibited the lysis of p20 and the secretion of p17 and TNF‐α in the supernatant. The results suggested that DP inhibited the lysis of p20 and the secretion of p17 in a dose‐dependent manner but had no effect on the production of the inflammasome‐independent cytokine TNF‐α (Figure 5H–S). Our experimental results demonstrated that DP treatment indeed blocked ATP/Nigericin‐induced p20 cleavage and p17 secretion.

ASC oligomerization is an essential event in NLRP3 inflammasome assembly. Next, we explored the effect of DP on ASC oligomerization during inflammasome assembly. First, we examined the changes in the oligomerization of ASC proteins in the NLRP3 inflammasome activated by ATP/Nigericin after 12 h of DP treatment of BMDMs at the protein level (Figure 5T,V). Second, we tested the extent of ASC oligomerization after LPS initiation of the NLRP3 inflammasome and 1 h of DP treatment of BMDMs with ATP/Nigericin activation of the inflammasome (Figure 5U,W). The results showed that DP blocked ASC oligomerization in a dose‐dependent manner. According to the above experimental results, we found that DP suppressed ATP/Nigericin‐induced ASC oligomerization (Figure 5T,W), suggesting that DP acts upstream of ASC oligomerization, thereby inhibiting NLRP3 activation. NLRP3 inflammasome activation triggers mitochondrial damage, leading to decreased mitochondrial membrane potential and mtROS production, acting as a central trigger of NLRP3 inflammasome activation. BMDMs were treated with various doses of DP for a duration of 12 h. Subsequently, BMDMs were primed with LPS and activated with ATP. Our results showed that the mitochondrial membrane potential was significantly reversed and that mtROS production was inhibited under different concentrations of DP (Supporting Information Figures S7 & S8). Moreover, LPS‐induced BMDMs were treated with different doses of DP and then stimulated with ATP/Nigericin. The experimental results showed that DP blocked ATP/Nigericin‐induced mitochondrial membrane potential decreases and mtROS production in BMDMs (Supporting Information Figures S7 & S8). Moreover, we examined the impact of DP on CMPK2 levels. The experimental results showed that DP dose‐dependently inhibited the protein expression of CMPK2 (Supporting Information Figure S9A–F), indicating that DP is a multi‐functional inhibitor of CMPK2. Accordingly, we further examined the changes in the RNA level of CMPK2 after DP treatment. As expected, DP decreased the mRNA expression level of *Cmpk2* in a dose‐dependent manner (Supporting Information Figure S9G–J). In summary, these results demonstrated that DP can block mitochondrial damage to a certain extent. In short, these results indicate DP‐mediated modulation of NLRP3 inflammasome activation.

### 3.6 CMPK2 plays a fundamental role in DP modulation of NLRP3 inflammasome activation

**FIGURE 6:**
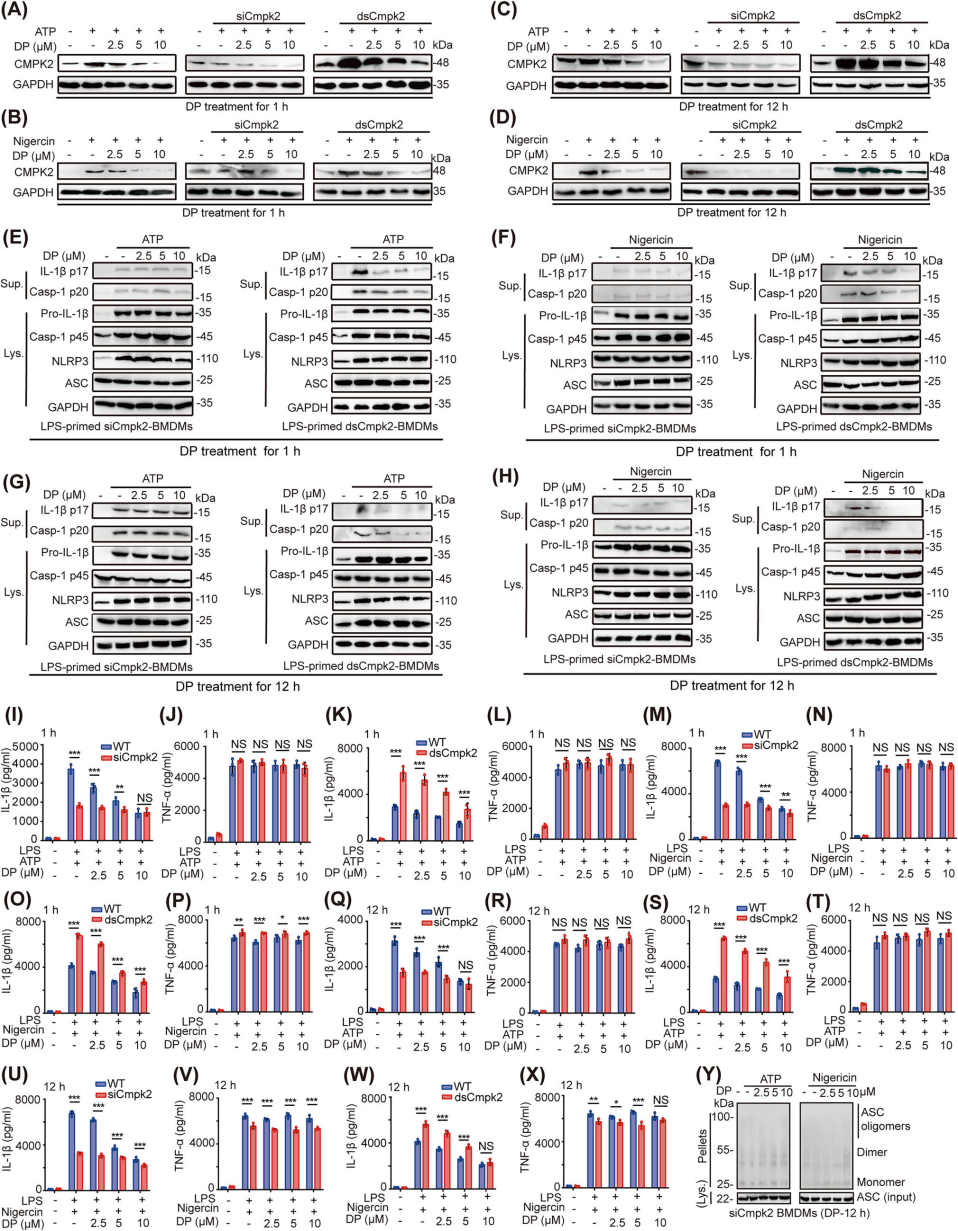
DP inhibits NLRP3 inflammasome activation via CMPK2. (A–D) Immunoblot analysis of CMPK2 in WT or specific sienna‐transduced or *dsCmpk2*‐transduced BMDMs with ATP/Nigericin‐activated inflammasomes. (E) Immunoblot analysis of the indicated proteins in Lys. and Sup. from specific *siCmpk2*‐transduced or *dsCmpk2*‐transduced BMDMs treated with DP for 1 h and then stimulated with ATP for 45 min. (F) LPS‐primed *siCmpk2*‐transduced or *dsCmpk2*‐transduced BMDMs treated with different concentrations (2.5, 5 and 10 μM) of DP for 1 h, followed by stimulation with 10 μM Nigericin for 45 min. Immunoblot analysis of the indicated proteins in Lys. and Sup. (G) Immunoblot analysis of the indicated proteins in Lys. and Sup. from specific *siCmpk2*‐transduced or *dsCmpk2*‐transduced BMDMs treated with DP for 12 h and then stimulated with ATP for 45 min. (H) BMDMs were treated with various doses of DP (2.5, 5 10 μM) for 12 h, and then LPS‐primed BMDMs were stimulated with 10 μM Nigericin. Immunoblot analysis of the indicated proteins in Lys. and Sup. (I, J, M and N) LPS‐primed *siCmpk2*‐transduced BMDMs treated with different concentrations (2.5, 5 and 10 μM) of DP for 1 h, followed by stimulation with 4 mM ATP or 10 μM Nigericin for 45 min for IL‐1β secretion and TNF‐α production in Sup.  (n = 3). (K, L, O and P) LPS‐primed *dsCmpk2*‐transduced BMDMs treated with different concentrations (2.5, 5 and 10 μM) of DP for 1 h, followed by stimulation with 4 mM ATP or 10 μM Nigericin for 45 min for IL‐1β secretion and TNF‐α production in Sup. (n = 3). (Q–X) BMDMs were treated with various doses of DP (2.5, 5 and 10 μM) for 12 h, and then LPS‐primed *siCmpk2*‐transduced or *dsCmpk2*‐transduced BMDMs were stimulated with 4 mM ATP or 10 μM Nigericin. IL‐1β secretion (Q, S, U and W) and TNF‐α production (R, T, V and X) in Sup. of BMDMs (n = 3). (Y) Immunoblot analysis of ASC oligomerization in lysates of specific *siCmpk2*‐transduced BMDMs treated with various doses (upper lanes) of DP for 12 h, and then LPS‐primed BMDMs were stimulated with 4 mM ATP or 10 μM Nigericin, input, or Sup. Statistics were analysed using an unpaired Student's *t* test: *p < .05; **p < .01; ***p < .001. NS, not significant. For (I–X), data are represented as mean ± SD.

Cellular targets are the molecular basis by which DP regulates the NLRP3 inflammasome. To explore the potential role played by CMPK2 in NLRP3 inflammasome activation progression, we knocked down CMPK2 with a specific sienna to characterize the function of CMPK2 in wild‐type BMDMs (Figure 6A–D). Immunoblot analysis of inflammasome sub‐unit expression in lysates of WT/*siCmpk2* BMDMs stimulated with LPS and then treated with different doses of DP for 1 h or WT/*siCmpk2* BMDMs treated with DP for 12 h and then stimulated with LPS, followed by inflammasome activation with ATP/Nigericin. The experimental results confirmed that, compared with wild‐type BMDMs, CMPK2‐deficient macrophages exhibited reduced caspase‐1 activation and inhibition of IL‐1β maturation (Figure 6E–H). ELISAs performed with IL‐1β and TNF‐α in supernatants from LPS‐primed WT or *siCmpk2* BMDMs that had been activated with ATP/Nigericin with or without DP (2.5, 5 or 10 μM) revealed that CMPK2‐deficient macrophages lost the ability to suppress subsequent IL‐1β production (Figure 6I,M,Q and U). It is worth noting that the above experimental results confirmed that regardless of whether BMDMs were treated with DP for 12 or 1 h, after ATP/Nigericin activated the NLRP3 inflammasome, there was no significant change in the release of TNF‐α in the supernatant (Figure 6J,N,R and V). Additionally, the results confirmed that DP suppressed ASC oligomerization (Figure 5T–W). Subsequently, an oligomerization assay was performed after CMPK2 was knocked down in BMDMs. We found that LPS‐induced ASC oligomerization was significantly blocked in specific *siCmpk2*‐transduced BMDMs. Meanwhile, our results indicated that ASC oligomerization was not significantly altered after treatment of specific *siCmpk2*‐transduced BMDMs with different concentrations of DP (Figure 6Y). This result indicates that the oligomerization of the ASC protein was completely inhibited by activation of the NLRP3 inflammasome after CMPK2 was knocked down in BMDMs.

CMPK2 plasmids were transfected into BMDMs to characterize the function of CMPK2 (Figure 6A–D). BMDMs were primed with LPS, followed by DP treatment or no treatment, and then BMDMs were stimulated with ATP/Nigericin. Western blot analysis showed that CMPK2 overexpression significantly promoted p20 secretion and p17 maturation compared with WT BMDMs (Figure 6E–F). After CMPK2 was overexpressed in BMDMs, the NLRP3 inflammasome was activated, and the release of the cytokines IL‐1β and TNF‐α in the supernatant of BMDMs was detected by ELISA. As expected, the experimental results of this experiment also confirmed that the inhibitory effect of DP on the cytokine IL‐1β was reversed after the addition of CMPK2 to BMDMs (Figure 6K,O). Similarly, after overexpression of CMPK2, the effect of DP on the inflammatory factor TNF‐α in the supernatant was negligible (Figure 6L,P). Then, these specific CMPK2 plasmid‐transduced BMDMs were treated with DP, subsequently stimulated with LPS and then activated with ATP/Nigericin. Immunoblot analysis was performed to investigate IL‐1β maturation and caspase‐1 activation, and the results indicated a dramatically reversed trend compared with BMDMs with CMPK2 knockdown (Figure 6G,H). We simultaneously examined the release of the cytokines IL‐1β and TNF‐α in the supernatant of BMDMs after overexpression of the CMPK2 plasmid, and the results showed that the release of the cytokine IL‐1β was significantly higher in the cell supernatant than in WT BMDMs (Figure 6S,W). Additionally, the release of IL‐1β into the supernatant was found to be significantly down regulated compared to that in BMDMs with CMPK2 knockdown. Moreover, after overexpression of CMPK2 in BMDMs, DP had no intervening effect on TNF‐α release into the supernatant (Figure 6T,X). Taken together, these experimental data suggest that CMPK2 is an important transport hub in the DP‐mediated regulation of NLRP3 inflammasome activation.

### 3.7 DP exerts beneficial effects in a mouse model of sepsis

**FIGURE 7:**
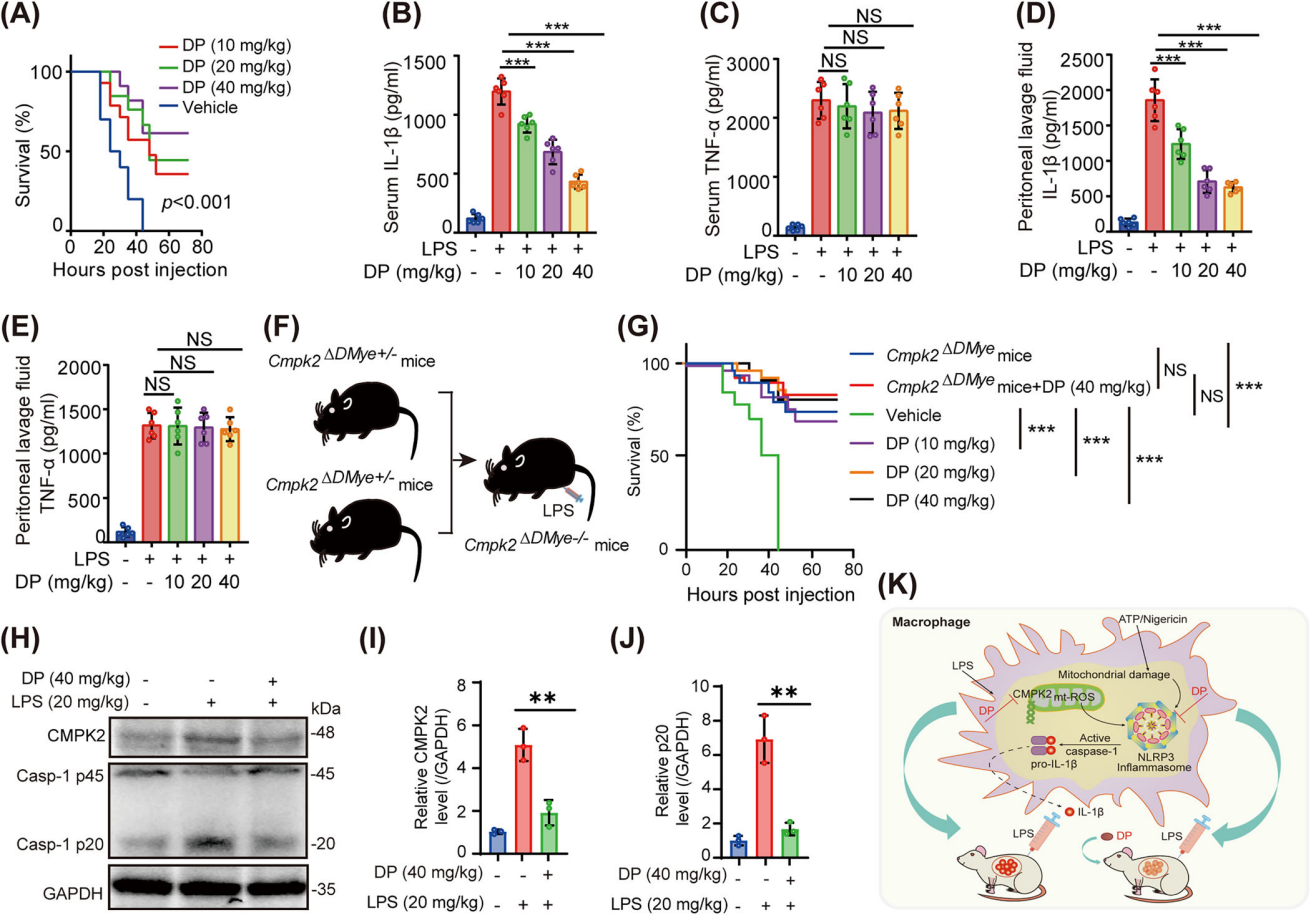
DP attenuates LPS‐induced septic shock in mice. (A) Eight‐week‐old C57BL/6 male mice were treated with vehicle or DP (10, 20 and 40 mg/kg), and 2 h later, they were i.p. injected with LPS (20 mg/kg). The survival of the mice was monitored for 72 h (n = 10/group). (B–E) Mice were treated with vehicle or DP (10, 20 and 40 mg/kg) for 2 h and then i.p. injected with LPS (20 mg/kg) for 6 h (n = 6/group). IL‐1β (B) and TNF‐α (C) in serum and IL‐1β (D) and TNF‐α (E) in peritoneal lavage fluid were analysed by ELISA. (F) Generated *Cmpk2^∆DMye^* mice. (G) Eight‐week‐old male *Cmpk2^∆DMye^* mice were treated with vehicle or DP (40 mg/kg), and 2 h later, they were i.p. injected with LPS (20 mg/kg). The survival of the mice was monitored for 72 h (n = 11/group). (H) DP blocked CMPK2 and caspase‐1 expression in vivo. Mice were treated with vehicle or DP (40 mg/kg) for 2 h and then i.p. injected with LPS (20 mg/kg) for 6 h. CMPK2 and caspase‐1 expression were detected by western blotting analysis of myeloid tissue from mice. GAPDH was used as a loading control. (I–J) Statistical analysis of the relative protein contents in (H). (K) Schematic summary of the role of DP in the regulation of sepsis via blockade of the LPS‐induced CMPK2 pathway in macrophages. Statistics were analysed using an unpaired Student's *t* test and log‐rank test: * *p* < .05; **p < .01; ***p < .001. NS, no significance. For (B–D, I and J), data are represented as mean ± SD.

Since DP inhibits NLRP3 inflammasome activation via CMPK2 in vitro, we subsequently analysed the functions exhibited by DP in vivo. We examined whether DP inhibited the NLRP3 inflammasome in vivo. IL‐1β production in a mouse model of septic shock is dependent on the activation of the NLRP3 inflammasome.^7^, ^12^, ^40^, ^41^ At the beginning of the experiment, we first monitored the interventional effect of DP on the survival of mice with acute septic shock (Figure 7A), and the results suggested that different concentrations of DP suppressed the lethal inflammation caused by NLRP3 inflammasome activation. Subsequently, we tested whether DP blocked the release of inflammatory cytokines in the serum and peritoneal lavage fluid of septic mice. First, mice were pre‐treated with DP for 2 h, and then, IL‐1β and TNF‐α levels were evaluated after 6 h of LPS treatment. As expected, the results clearly showed that DP reduced IL‐1β production but did not affect TNF‐α production (Figure 7B–E). To verify the decisive role played by the target protein CMPK2 in septic mice, we observed the survival of *Cmpk2^∆DMye^* mice (mice with myeloid‐specific ablation of CMPK2) for 72 h (Figure 7F). Surprisingly, the results of this part of the experiment were exactly what we expected, and the experimental data revealed that the survival rate of *Cmpk2^∆DMye^* mice increased significantly (Figure 7G). However, administration of DP in *Cmpk2^∆DMye^* mice did not further improve the survival rate (Figure 7G). These results suggest that DP plays a curative role in septic mice in a manner dependent on CMPK2 in macrophages. In addition, we explored whether DP blocks CMPK2 and caspase‐1 expression in vivo. Briefly, mice were treated with vehicle or DP for 2 h, followed by intraperitoneal injection (i.p.) of LPS for 6 h. Expression of CMPK2 and caspase‐1 was visualized by western blot analysis of mouse myeloid tissues (Figure 7H), indicating that DP still markedly blocked CMPK2 expression in vivo (Figure 7I). Moreover, our data also elucidated that DP prevents the increase in activated caspase‐1 in an experimental sepsis mouse model (Figure 7J).

Next, we observed that DP treatment reversed major changes in liver, lung and kidney morphology in septic shock mice (Supporting Information Figures S10, S11 & S12). Histopathological analysis of the liver, lung and kidney revealed that LPS‐induced inflammatory infiltration and fibrosis (haematoxylin and eosin staining, Sirius red staining and Masson staining) were distinctly alleviated after DP treatment. This further indicated that DP could inhibit the activation of the NLRP3 inflammasome in vivo and had a protective effect against sepsis in mice (Figure 7K). Before and after the outbreak of sepsis, the effects of using DP, such as the survival curve and phenotype index, were measured. The related results are shown in Supporting Information (Supporting Information Figure S13). We found that whether mice were treated with DP first or after the sepsis outbreak, DP significantly improved the survival rate of septic mice. To monitor the effects of DP after an outbreak of sepsis, we divided the trial into four groups: normal, model, administration of 1 h (DP before injection of LPS), and administration of 2 h (DP after injection of LPS). The results showed that the body weight, body temperature and heart rate of the normal group were significantly different from those of the model group, while there was no significant difference between the model group and the drug administration groups. However, for the establishment of an acute sepsis model in mice, we monitored the blood pressure changes of mice between different groups with a BP‐2010A instrument. As far as the experimental results are available, we have not captured significant differences between the groups.

Given that we have constructed an acute sepsis model, the phenotypic explosion of the disease in the organism is extremely rapid and severe. Therefore, a BP‐2010A instrument was used to detect the heart rate and blood pressure of mice non‐invasively, but considering the large fluctuation range of heart rate and blood pressure monitored by this instrument. The BIOPAC System‐MP160 was used to more accurately monitor heart rate and blood pressure in septic mice. The results showed that DP could improve hypotension in septic mice, but no significant difference was observed in heart rate (Supporting Information Figure S14). By observing the changes in blood pressure from day 2 to 3 in the model group and the two treatment groups, the experimental data suggested that early administration of DP was more effective in the treatment of septic mice. In brief, DP is a natural active component extracted from palm fruit, and our results suggest that this active component regulates the NLRP3 inflammasome through the LPS‐induced CMPK2 pathway in macrophages. Overall, the experimental results clarify that DP has tremendous development value for novel therapies targeting NLRP3‐driven diseases such as sepsis.

## 4 DISCUSSION

Dragon's blood has a variety of pharmacological effects, including immunomodulatory, anti‐inflammatory, anti‐diarrhoeal, anti‐bacterial, anti‐viral, anti‐oxidant and anti‐cancer effects.^42^ However, its mechanism of action is unknown, limiting its clinical use. As one of the main active extracts of dragon's blood, DP is present in the form of a specific salt called dracorhodin perchlorate. Here, the purpose of this work was to develop a two‐step chemical tracking strategy to identify and evaluate a naturally derived active small molecule inhibitor of CMPK2, DP, which significantly inhibits NLRP3 inflammasome activation by blocking the LPS‐induced CMPK2 pathway, suggesting a significant advantage of DP in the treatment of NLRP3‐driven related diseases.

A question worth pondering is how the active ligand DP targets CMPK2 to inhibit the occurrence of inflammatory responses. Our results demonstrate that DP conformational intervention of the Lys265 sites of CMPK2. Nevertheless, unlike other nucleotide kinases, no CMPK2 structure is available thus far. To study the mode of DP binding to human CMPK2, we tried to determine the structure of human CMPK2 complexed with DP. Nevertheless, a series of attempts to co‐crystallize the *N*‐terminal truncated human CMPK2 protein with the inhibitor DP were unsuccessful. According to the results of the molecular dynamics simulation, it is speculated that the high flexibility of CMPK2 restrains its crystal packing. Further studies need to be conducted. Interestingly, K320 is far from the binding pocket of CMPK2, and it is surprising that the binding of DP disrupted its reactivity. The reason for this conformational intervention may be that DP triggers the rotation of the kinase activation loop and the formation of an allosteric interaction network.^35^ Hence, the conformational effect of DP on K320 might be attributed to the involvement of K320 in this allosteric interaction network. The mutant CMPK2^Lys282^ was expressed and purified. The *K_d_* values of the mutants and DP were detected by MST technology, and no binding between DP and CMPK2 was found after Lys282 was mutated. In addition, we detected the activity of the mutant protein using ADP‐GLO, and the results showed that DP still had a slight inhibitory effect on the kinase activity of the mutant at K282. In view of this experimental phenomenon, further experimental exploration is needed.

In this study, we confirmed that DP blocks the LPS‐induced CMPK2 pathway to inhibit NLRP3 inflammasome assembly. During the process of target gene interference in BMDMs, we were surprised to find that DP significantly reduced the expression of the target protein CMPK2 in BMDMs. We designed subsequent experiments and examined the impact of DP on CMPK2 levels via two different DP treatments. BMDMs were treated with DP before or after LPS stimulation, and the NLRP3 inflammasome was activated using ATP/Nigericin. Then, the protein expression of CMPK2 was detected by immunoblot analysis. The experimental results showed that DP dose‐dependently inhibited the protein expression of CMPK2, indicating that DP is a multi‐functional inhibitor of CMPK2. DP not only inhibited the activity of CMPK2 kinase in vitro but also blocked the expression of CMPK2 protein in vivo. Accordingly, we further examined the changes in the RNA level of the intra‐cellular kinase CMPK2 after DP intervention. After ATP/Nigericin activation of the inflammasome, DP decreased the mRNA expression level of CMPK2 in a dose‐dependent manner. This result is very shocking to us because it suggests that the in vivo effect of DP depends on both direct binding of CMPK2 and a reduction in CMPK2 expression. However, the targets of DP that reduce the mRNA expression levels of CMPK2 need to be further explored.

Mitochondrial damage, which is characterized by the excessive generation of reactive oxygen species and a decrease in mitochondrial membrane potential, is identified as an upstream signalling event of inflammasome activation. Basic scientific research has confirmed that mitochondrial dysfunction and potassium efflux are two major mechanisms of activation of the NLRP3 inflammasome. According to the present experimental results, DP inhibits NLRP3 inflammasome activation by blocking mitochondrial damage. Whether this effect specifically inhibits NLRP3 inflammasome activation requires further investigation. The main objective of this study was to establish integrated high‐throughput chemical tracking strategies to rapidly characterize the active ligand of the target protein CMPK2. In addition, the results from the previous section confirmed that DP suppressed Nigericin/ATP‐induced ASC oligomerization. In this section, there is some confusion about exactly how DP inhibits NLRP3 inflammasome activation. Does DP inhibit NLRP3 inflammasome activation by inhibiting CMPK2 or by inhibiting ASC oligomerization? Subsequently, CMPK2 was knocked down in BMDMs and an oligomerization assay was performed. The experimental results indicate that the oligomerization of the ASC protein was completely inhibited by activation of the NLRP3 inflammasome after CMPK2 was knocked down in BMDMs. A study reported that *Cmpk2^∆Mye^* mice also had fewer alveolar macrophages with ASC specks, indicating decreased activation of the NLRP3 inflammasome,^26^ confirming the accuracy of our experimental results.

This study explored a novel agent that specifically targets the LPS‐induced CMPK2 kinase pathway with the aim of providing more possibilities for alleviating inflammation‐related diseases. We have developed an affinity MS strategy combined with quantitative lysine reactivity profiling to discover novel CMPK2 ligands from a range of natural product extracts for the purpose of intervening in inflammation. First, recombinant CMPK2 kinase was purified by a conventional eukaryotic expression purification system, and the kinase activity of recombinant CMPK2 was detected by the ADP‐GLO assay. Then, we used this strategy to identify a naturally sourced small molecule, DP, as the first CMPK2 inhibitor. Finally, our overall experimental results suggest that DP regulates the NLRP3 inflammasome through the LPS‐induced CMPK2 kinase pathway. These results indicate that DP could serve as a precursor in the development of new therapeutics for sepsis.

As sepsis is a systemic disease involving several organs, the pathogenesis of sepsis remains incompletely elucidated, and patients currently die even after admission. Current clinical guidelines can help implement effective management, restore adequate cell perfusion through early appropriate anti‐bacterial therapy, and enable timely source control to improve the prognosis of patients with sepsis. Although many clinical trials are underway, there is currently no FDA‐approved drug for the treatment of sepsis. Therefore, additional focused research is required to strengthen our understanding of the basic pathophysiology and causes of death in sepsis. Herein, we found the inhibitor DP of CMPK2 for the first time by using an affinity MS strategy combined with quantitative lysine reactivity profiling. DP acted as an inhibitor and exerted a strong anti‐inflammatory effect by regulating the NLRP3 inflammasome mediated via the LPS‐induced CMPK2 pathway in macrophages. Specifically, DP suppressed CMPK2 kinase, thereby blocking NLRP3 inflammasome activation and abrogating IL‐1β release. Strikingly, DP significantly attenuated LPS‐induced sepsis by restricting CMPK2, but its effect was weakened in *Cmpk2* ^∆DMye^ mice. This suggests the potential of DP for the treatment of sepsis.

## CONFLICT OF INTEREST STATMENT

The authors declare no conflict of interest.

## Supporting information

Supporting Information

Supporting Information

## Data Availability

The data that support the findings of this study are available from the corresponding author upon reasonable request.

## References

[1] Kotas ME , Medzhitov R . Homeostasis, inflammation, and disease susceptibility. Cell. 2015;160:816‐827. doi:10.1016/j.cell.2015.02.010 25723161 PMC4369762

[2] Zhong Z , Liang S , Sanchez‐Lopez E , et al. New mitochondrial DNA synthesis enables NLRP3 inflammasome activation. Nature. 2018;560:198‐203. doi:10.1038/s41586-018-0372-z 30046112 PMC6329306

[3] Karin M , Clevers H . Reparative inflammation takes charge of tissue regeneration. Nature. 2016;529(7586):307‐315. doi:10.1038/nature17039 26791721 PMC5228603

[4] Corcoran SE , Halai R , Cooper MA . Pharmacological inhibition of the NOD‐like receptor family pyrin domain containing 3 inflammasome with mcc950. Pharmacol Rev. 2021;73:968‐1000. doi:10.1124/PHARMREV.120.000171 34117094

[5] Coll RC , Hill JR , Day CJ , et al. MCC950 directly targets the NLRP3 ATP‐hydrolysis motif for inflammasome inhibition. Nat Chem Biol. 2019;15:556‐559. doi:10.1038/s41589-019-0277-7 31086327

[6] Lee E , Hwang I , Park S , et al. MPTP‐driven NLRP3 in flammasome activation in microglia plays a central role in dopaminergic neurodegeneration. Cell Death Differ. 2019;26(2):213‐228. doi:10.1038/s41418-018-0124-5 29786072 PMC6329843

[7] Jiang H , He H , Chen Y , et al. Identification of a selective and direct NLRP3 inhibitor to treat inflammatory disorders. J Exp Med. 2017;214(11):3219‐3238. 29021150 10.1084/jem.20171419 PMC5679172

[8] Tang T , Gong T , Jiang W , Zhou R . GPCRs in NLRP3 inflammasome activation, regulation, and therapeutics. Trends Pharmacol Sci. 2018;39:798‐811. doi:10.1016/j.tips.2018.07.002 30054020

[9] Rathkey JK , Zhao J , Liu Z , et al. Chemical disruption of the pyroptotic pore‐forming protein gasdermin D inhibits inflammatory cell death and sepsis. Sci Immunol. 2018;3:eaat2738. doi:10.1126/sciimmunol.aat2738 30143556 PMC6462819

[10] Xia S , Zhang Z , Magupalli VG , et al. Gasdermin D pore structure reveals preferential release of mature interleukin‐1. Nature. 2021;593:607‐611. doi:10.1038/s41586-021-03478-3 33883744 PMC8588876

[11] Niu T , De Rosny C , Chautard S , et al. NLRP3 phosphorylation in its LRR domain critically regulates inflammasome assembly. Nat Commun. 2021;12:1‐15. doi:10.1038/s41467-021-26142-w 34615873 PMC8494922

[12] Huang Y , Jiang H , Chen Y , et al. Tranilast directly targets NLRP3 to treat inflammasome‐driven diseases. EMBO Mol Med. 2018;10:1‐15. doi:10.15252/emmm.201708689 29531021 PMC5887903

[13] Gross O , Poeck H , Bscheider M , et al. Syk kinase signalling couples to the Nlrp3 inflammasome for anti‐fungal host defence. Nature. 2009;459:433‐436. doi:10.1038/nature07965 19339971

[14] Dinarello CA , Van der Meer JWM . Treating inflammation by blocking interleukin‐1in humans. 2013;25:469‐484. doi:10.1016/j.smim.2013.10.008 PMC3953875 24275598

[15] Riley JL , Gordan VV , Rouisse KM , McClelland J , Gilbert GH . Gender differences in practice patterns for diagnosis and treatment of dental caries: findings from the dental PBRN. J Am Dent Assoc. 2011;142:429‐440. doi:10.1038/nrd3800.Treating 21454850 PMC3079556

[16] Nowarski R , Jackson R , Gagliani N , et al. Epithelial IL‐18 equilibrium controls barrier function in colitis. Cell. 2015;163:1444‐1456. doi:10.1016/j.cell.2015.10.072 26638073 PMC4943028

[17] Netea MG , Van De Veerdonk FL , Van Der Meer JWM , Dinarello CA , Joosten LAB . Inflammasome‐independent regulation of IL‐1‐family cytokines. Annu Rev Immunol. 2015;33:49‐77. doi:10.1146/annurev-immunol-032414-112306 25493334

[18] Cocco M , Pellegrini C , Martínez‐Banaclocha H , et al. Development of an acrylate derivative targeting the NLRP3 inflammasome for the treatment of inflammatory bowel disease. J Med Chem. 2017;60:3656‐3671. doi:10.1021/acs.jmedchem.6b01624 28410442

[19] Goldberg EL , Asher JL , Molony RD , et al. β‐hydroxybutyrate deactivates neutrophil NLRP3 inflammasome to relieve gout flares. Cell Rep. 2017;18:2077‐2087. doi:10.1016/j.celrep.2017.02.004 28249154 PMC5527297

[20] Chen L , Huang CF , Li YC , et al. Blockage of the NLRP3 inflammasome by MCC950 improves anti‐tumor immune responses in head and neck squamous cell carcinoma. Cell Mol Life Sci. 2018;75:2045‐2058. doi:10.1007/s00018-017-2720-9 29184980 PMC11105265

[21] Dempsey C , Rubio Araiz A , Bryson KJ , et al. Inhibiting the NLRP3 inflammasome with MCC950 promotes non‐phlogistic clearance of amyloid‐β and cognitive function in APP/PS1 mice. Brain Behav Immun. 2017;61:306‐316. doi:10.1016/j.bbi.2016.12.014 28003153

[22] Xu Y , Johansson M , Karlsson A . Human UMP‐CMP kinase 2, a novel nucleoside monophosphate kinase localized in mitochondria. J Biol Chemist. 2008;283:1563‐1571. doi:10.1074/jbc.M707997200 17999954

[23] Zhao M , Su HZ , Zeng YH , et al. Loss of function of CMPK2 causes mitochondria deficiency and brain calcification. Cell Discov. 2022;8:128. doi:10.1038/s41421-022-00475-2 36443312 PMC9705363

[24] Liu W , Chen B , Li C , et al. Identification of fish CMPK2 as an interferon stimulated gene against SVCV infection. Fish Shellfish Immunol. 2019;92:125‐132. doi:10.1016/j.fsi.2019.05.032 31125665

[25] El‐Diwany R , Soliman M , Sugawara S , et al. CMPK2 and BCL‐G are associated with type 1 interferon–induced HIV restriction in humans. Sci Adv. 2018;4:1‐12. doi:10.1126/sciadv.aat0843 PMC6070316 30083606

[26] Xian H , Liu Y , Rundberg Nilsson A , et al. Metformin inhibition of mitochondrial ATP and DNA synthesis abrogates NLRP3 inflammasome activation and pulmonary inflammation. Immunity. 2021;54:1463‐1477. doi:10.1016/j.immuni.2021.05.004 e11 34115964 PMC8189765

[27] Zhang W , Chen Y , Jiang H , et al. Integrated strategy for accurately screening biomarkers based on metabolomics coupled with network pharmacology. Talanta. 2020;211:120710. doi:10.1016/j.talanta.2020.120710 32070601

[28] Thornton JM , Laskowski RA , Borkakoti N . AlphaFold heralds a data‐driven revolution in biology and medicine. Nat Med. 2021;27:1666‐1669. doi:10.1038/s41591-021-01533-0 34642488

[29] Jumper J , Evans R , Pritzel A , et al. Highly accurate protein structure prediction with AlphaFold. Nature. 2021;596:583‐589. doi:10.1038/s41586-021-03819-2 34265844 PMC8371605

[30] Ma J , Lu Y , Wu D , et al. Ligand identification of the adenosine A2A receptor in self‐assembled nanodiscs by affinity mass spectrometry. Analyt Methods. 2017;9:5851‐5858. doi:10.1039/c7ay01891f

[31] Zhang B , Zhao S , Yang D , et al. A novel G protein‐biased and subtype‐selective agonist for a G protein‐coupled receptor discovered from screening herbal extracts. ACS Cent Sci. 2020;6:213‐225. doi:10.1021/acscentsci.9b01125 32123739 PMC7047268

[32] Senior AW , Evans R , Jumper J , et al. Improved protein structure prediction using potentials from deep learning. Nature. 2020;577:706‐710. doi:10.1038/s41586-019-1923-7 31942072

[33] Adler J , Wu Z , Green T , et al. Highly accurate protein structure prediction for the human proteome. Nature. 2021;596(7873):590‐596. doi:10.1038/s41586-021-03828-1 34293799 PMC8387240

[34] Liu Z , Zhang W , Sun B , et al. Probing conformational hotspots for the recognition and intervention of protein complex by lysine reactivity profiling. Chem Sci. 2021;12(4):1451‐1457. doi:10.1039/d0sc05330a PMC8179027 34163908

[35] Chen J , Wang A , Liu B , et al. Quantitative lysine reactivity profiling reveals conformational inhibition dynamics and potency of aurora a kinase inhibitors. Anal Chem. 2019;91:13222‐13229. doi:10.1021/acs.analchem.9b03647 31525957

[36] Ostermann N , Schlichting I , Brundiers R , et al. Insights into the phosphoryltransfer mechanism of human thymidylate kinase gained from crystal structures of enzyme complexes along the reaction coordinate. Structure. 2000;8(6):629‐642. 10873853 10.1016/s0969-2126(00)00149-0

[37] Segura‐Peña D , Sekulic N , Ort S , Konrad M , Lavie A . Substrate‐induced conformational changes in human UMP/CMP kinase. Journal of Biological Chemistry. 2004;279:33882‐33889. 15163660 10.1074/jbc.M401989200

[38] Schlichting I , Reinstein J . Structures of Active Conformations of UMP Kinase from Dictyostelium discoideum Suggest Phosphoryl Transfer Is Associative. 1997;2960:9290‐9296. 10.1021/bi970974c 9280438

[39] Scheffzek K , Kliche W , Wiesmüller L , Reinstein J . Crystal structure of the complex of UMP/CMP kinase from Dictyostelium discoideum and the bisubstrate inhibitor P1‐(5′‐adenosyl) P5‐(5′‐uridyl) pentaphosphate (UP5A) and Mg2+ at 2.2 Å: Implications for water‐mediated specificity. Biochemistry. 1996;35:9716‐9727. 8703943 10.1021/bi960642s

[40] Liu H , Zhan X , Xu G , et al. Cryptotanshinone specifically suppresses NLRP3 inflammasome activation and protects against inflammasome‐mediated diseases. Pharmacol Res. 2021;164:105384. doi:10.1016/j.phrs.2020.105384 33352229

[41] He H , Jiang H , Chen Y , et al. Oridonin is a covalent NLRP3 inhibitor with strong anti‐inflammasome activity. Nat Commun. 2018;9:1‐12. doi:10.1038/s41467-018-04947-6 29959312 PMC6026158

[42] Jiang X , Liu L , Qiao L , et al. Dracorhodin perchlorate regulates fibroblast proliferation to promote rat's wound healing. J Pharmacol Sci. 2018;136:66‐72. doi:10.1016/j.jphs.2017.12.003 29428295

