# Supplementary material for: Dracorhodin targeting CMPK2 attenuates inflammation: A novel approach to sepsis therapy
Source: Clin Transl Med. 2023 Oct 20;13(10):e1449. doi: 10.1002/ctm2.1449 (PMC10587737; doi:10.1002/ctm2.1449)
Supplement: Supplementary file 1 — Supporting Information [file CTM2-13-e1449-s002.docx]

**Supplementary Materials**

**Dracorhodin targeting CMPK2 attenuates inflammation: A novel approach to sepsis therapy**

Wendan Zhang^1,2,7^, Honghong Jiang^1,2,7^, Pengli Huang^1,7^, Gaosong Wu^1^, Qun Wang^1^, Xin Luan^1^, Hongwei Zhang^1^, Dianping Yu^1^, Hongru Wang^1^, Dong Lu^1^, Haonan Wang^1,8^, Huazhang An^3,8^, Sanhong Liu^1,8^, Weidong Zhang^1,4,5,6,8^

^1^ Shanghai Frontiers Science Center of TCM Chemical Biology, Institute of Interdisciplinary Integrative Medicine Research, Shanghai University of Traditional Chinese Medicine, Shanghai, 201203, China

^2^ Faculty of Pediatrics, the Chinese PLA General Hospital, National Engineering Laboratory for Birth defects prevention and control of key technology, Beijing Key Laboratory of Pediatric Organ Failure, Beijing, China

^3^ Shandong Provincial Key Laboratory for Rheumatic Disease and Translational Medicine, The First Affiliated Hospital of Shandong First Medical University & Shandong Provincial Qianfoshan Hospital, Jingshi Road 16766, Jinan, Shandong 250014, China

^4^ Department of Phytochemistry, School of Pharmacy, Second Military Medical University, Shanghai, 200433, China

^5^ The Research Center for Traditional Chinese Medicine, Shanghai Institute of Infectious Diseases and Biosecurity, Shanghai University of Traditional Chinese Medicine, Shanghai, 201203, China

^6^ Institute of Medicinal Plant Development, Chinese Academy of Medical Sciences and Peking Union Medical College, Beijing, 100193, China

^7^ These authors contributed equally to this work.

**^8^ Correspondence：**Weidong Zhang, Shanghai Frontiers Science Center of TCM Chemical Biology, Institute of Interdisciplinary Integrative Medicine Research, Shanghai University of Traditional Chinese Medicine, Shanghai, 201203, China. Department of Phytochemistry, School of Pharmacy, Second Military Medical University, Shanghai, 200433, China. The Research Center for Traditional Chinese Medicine, Shanghai Institute of Infectious Diseases and Biosecurity, Shanghai University of Traditional Chinese Medicine, Shanghai, 201203, China.

Email: [wdzhangy@hotmail.com](mailto:wdzhangy@hotmail.com)

Sanhong Liu, Shanghai Frontiers Science Center of TCM Chemical Biology, Institute of Interdisciplinary Integrative Medicine Research, Shanghai University of Traditional Chinese Medicine, Shanghai, 201203, China.

Email:[liush@shutcm.edu.cn](mailto:liush@shutcm.edu.cn)

Huazhang An, Shandong Provincial Key Laboratory for Rheumatic Disease and Translational Medicine, The First Affiliated Hospital of Shandong First Medical University & Shandong Provincial Qianfoshan Hospital, Jingshi Road 16766, Jinan, Shandong 250014, China.

Email: [anhz@immunol.org](mailto:anhz@immunol.org)

Haonan Wang, Shanghai Frontiers Science Center of TCM Chemical Biology, Institute of Interdisciplinary Integrative Medicine Research, Shanghai University of Traditional Chinese Medicine, Shanghai, 201203, China.

Email: [wanghaonan0611@126.com](mailto:wanghaonan0611@126.com).

- 1. **Reagents and animals**

**2.2.1 Reagents**

Ultrapure LPS (*E. coli* O111:B4) and ATP were obtained from Sigma-Aldrich. Nigericin was [obtain](javascript:;)ed from MedChemExpress. MitoSOX was purchased from Life Technologies. M-CSF was obtained from GenScript. The antibodies used for immunoblotting analysis were as follows: anti-mouse IL-1β (12426S, Cell Signaling Technologies), anti-mouse NLRP3 (AG-20B-0014-C100, Adipogen), anti-mouse ASC (AG-25b-0006-C100, Adipogen), anti-mouse caspase-1 (AG-20B-0042-C100, Adipogen), anti-mouse ASC (sc-514414, Santa Cruz) and anti-GAPDH (60004-1, Proteintech). Dracorhodin (DP, CAS NO.125536-25-6) was purchased from Standard. Ammonium acetate (NH_4_Ac, purity > 98%), acetic acid (HAc, purity > 98%), DL-dithiothreitol (DTT) and bovine serum albumin (BSA, purity > 98%, part# A1470) were provided by Sigma-Aldrich (St. Louis, MO, USA). Deionized water was prepared by a Milli-Q system (Millipore, Bedford, MA, USA). LC-MS grade formic acid, LC-MS grade methanol and HPLC-grade acetonitrile were purchased from Thermo Fisher Scientific (Sunnyvale, USA). 4-(2-hydroxyethyl)-1-piperazineethane-sulfonic acid (HEPES), dimethyl sulfoxide (DMSO), formaldehyde (CH_2_O), formaldehyde-D2 (CD_2_O), sodium cyanoborohydride (NaBH_3_CN), ammonium acetate (NH_4_Ac), guanidine hydrochloride, iodoacetamide (IAM), α-chymotrypsin and trifluoroacetic acid (TFA) were obtained from Sigma (St. Louis, MO, USA). MILLEX-GV Filter Unit 0.22 μm PVDF and Amicon Μltra-0.5 10K Centrifugal Filter Devices were purchased from Merck Millipore (Darmstadt, Germany). Protease inhibitor cocktail and 100× DMSO stock solution were provided by Shanghai Yisheng Biotechnology Company. This experiment was implemented with natural small molecule compounds, which were provided by Second Military Medical University.

- - 1. **Animals**

The C57BL/6J mice used in the studies were obtained from the Model Animal Research Center of Shanghai University of Traditional Chinese Medicine. All animals were housed under a 12-hr light/dark cycle at 22-24°C with unrestricted access to food and water for the duration of the experiment except during fasting tests. Myeloid-specific *Cmpk2*-deficient (*Cmpk2^DMye^*) mice were donated by Prof. Huazhang An from Shandong Provincial Key Laboratory for Rheumatic Disease and Translational Medicine. All animal experiments were approved by the Regional Ethics Committee of Shanghai University of Traditional Chinese Medicine.

- 1. **Dimethylation labeling of the CMPK2 complex and liquid chromatography-tandem MS**

**2.6.1 Dimethylation labeling of the CMPK2 complex**

Stable isotope labeling reactions were performed in the active state of the target protein samples. In short, the sample was split into two identical portions. One portion was incubated with DP, and the other was incubated with DMSO vehicle for 30 min at 37℃. Then, mass-differentiated isotopic labeling was performed with heavy (30 mM CD_2_O, 5 mM NaBH_3_CN) and light (30 mM CH_2_O, 5 mM NaBH_3_CN) labeling reagents. Active isotopic labeling was allowed to proceed for the indicated time, and this experiment allowed incubation for 15 min at 25℃ in the dark. Add 5 M NH_4_AC and react for 10 min at room temperature to stop the labeling reaction. Then, the two labeled samples were mixed together, denatured, and digested by chymotrypsin with a substrate/enzyme ratio of 1:50 at 30°C for 5 h. The digested peptide mixture was desalted on an MnoSpinTM C18 column (GLSciences Inc, JAP). The digested peptide samples were collected by centrifugation, lyophilized, and stored at -80℃ for quantitative proteomic analysis.

- - 1. **Liquid chromatography-tandem MS**

The digested peptides were detected using a Q Exactive HF-X mass spectrometer equipped with an Easy-nLC 1200 chromatography system (Thermo Fisher Scientific). The samples were loaded onto a C18 trap column (3 cm × 200 μm i.d.) and separated on a C18 capillary column (15 cm × 75 μm i.d.) at a flow rate of 300 nL/min. Aqueous solutions of 0.1% formic acid (solvent A) and 80% acetonitrile with 0.1% formic acid (solvent B) were used in reversed-phase binary gradient separation. The gradient separation was set as follows: 6% - 25% B (0.1% FA in acetonitrile), 35 min, 25% - 37% B, 7 min, 37% - 95% B, 2 min, 95 B, 9 min. MS was operated in data-dependent acquisition. The MS full scans were acquired in the range of m/z 375-1800 with an AGC target of 3e^6^ at 50 ms IT. The dd-MS^2^ spectra were collected using an orbitrap analyzer with a resolution of 15000, and the top 15 abundant ions in the full MS spectrum were subjected to CID with an isolation window of 1.6 *m/z*. The AGC of dd-MS^2^ settings was set at 1e^3^. Dynamic exclusion was enabled with an exclusion time of 30 s.

**2.8 Optimization of the chromatographic conditions**

To ensure a good chromatographic separation effect in a short analysis time, different chromatographic columns, mobile phase systems, elution programs, flow rates, column temperatures and injection volumes were further optimized. Using resolution, tailing coefficient and RTs as the examined parameters, the final optimized chromatographic separation conditions were performed on an Acquity UPLC BEH C18 column (1.7 μm, 2.1×100 mm, Waters, USA) at a flow rate of 0.3 ml/min maintained at 40°C, and the mobile phase was water-0.1% formic acid (A) and acetonitrile (B). The gradient elution procedure was as follows: 0-2 min, 5% B; 2-12 min, 5-30% B; 12-25 min, 30-90% B; 25-30 min, 90% B; equilibration for 5 min before injection, and the injection volume was 3 μl. Under these conditions, most peaks can be separated within 35 min.

- 1. **Culture and stimulation of bone marrow-derived macrophages**

Mouse bone marrow cells (isolated from the legs of mice) were cultured in DMEM supplemented with 20% FBS. BMDMs were generated by culturing mouse bone marrow cells in the presence of 20 ng/ml M-CSF-conditioned medium for 7 days. BMDMs were seeded in 6- or 48-well plates overnight in FBS-free DMEM. On day 2, after priming with ultrapure LPS (200 ng/ml) for 4 h, BMDMs (1 × 10^6^ cells/ml) were further stimulated with ATP (4 mM) or nigericin (10 μM) for 45 min. Supernatants and cell lysates were collected for ELISA and immunoblot analyses.

- 1. **Cell counting kit 8 assay and ELISA**

**2.12.1 Cell counting kit 8 assay**

To detect the viability of cells, a cell counting kit 8 (CCK-8) assay was performed. BMDMs were seeded at 1×10^6^ cells/ml in a 96-well plate and incubated overnight at 37℃. Then, we treated the cells with different concentrations of DP (0-160 μM) for 24 h, added CCK-8 reagent to the cell medium and incubated the cells for 1 h. Then, the optical density was measured at a wavelength of 450 nm.

- - 1. **ELISA**

The cytokines mouse IL-1β and TNF-α in supernatants from serum, cell culture, and tissue culture were assayed by ELISA kits (R&D Systems, Minneapolis, MN, USA) according to the manufacturer’s instructions.

- 1. **MST**

The MST assay is an emerging technique used to quantitatively elucidate binding. The binding parameter between CMPK2 and DP was measured using MST on a Monolith NT.115 system (Germany). CMPK2 was fluorescently labeled with the Monolith NT Protein Labeling Kit RED (L018, NanoTemper Technologies GmbH, Munich, Germany) according to the manufacturer’s instructions. The Monolith NT.115 system was used with Monolith NT.115 standard capillaries (M0-K022). MST assay buffer was used to dilute the labeled CMPK2 to 2 μM. Sixteen dilutions (1:1) of the ligand were prepared using the same buffer, starting at a concentration of 10 μM. An equivalent volume of the labeled CMPK2 was added to each of the dilutions and loaded into Monolith NT.115 standard treated capillaries (Nano Temper Technologies), which were used to measure the MST trace at a temperature of 27℃. The experimental parameters were adjusted to medium MST power and 40% LED/excitation power. The binding and dissociation constants (*K_d_*) were evaluated and calculated using M0-Affinity analysis software version 2.1.3 (Nano Temper Technologies) for the ligand.

**2.16 The effect of DP on cellular CMPK2 activity**

Primary BMDMs were generated by culturing mouse bone marrow cells in the presence of 20 ng/ml M-CSF conditioned medium for 7 days. BMDMs were seeded in 6-well plates overnight in FBS-free DMEM. After adhering to the wall, different concentrations of DP were added and treated for 12 hours. On day 2, after priming with ultrapure LPS (200 ng/ml) for 4 h, BMDMs (1 × 10^6^ cells/ml) were further stimulated with ATP (4 mM) or Nigericin (10 μM) for 45 min. Cell lysates were collected for ADP-Glo™ Kinase Assay (Promega, Madison, WI, USA).

- 1. **ASC oligomerization assay and immunoblot analysis**

**2.18.1 ASC oligomerization assay**

BMDMs were seeded at 1×10^6^ cells/ml in 6-well plates. After cell adherence, BMDMs were treated with different concentrations of DP. The following day, the medium was replaced, and the cells were primed with 200 ng/ml LPS for 4 h and then stimulated with Nigericin or ATP for 45 min. BMDMs were primed with 200 ng/ml LPS for 4 h. The cells were treated with DP for 1 h and then stimulated with Nigericin or ATP for 45 min. The supernatant was removed, the cells were rinsed in ice-cold PBS, and then, the cells were lysed with NP-40 for 30 min. Lysates were centrifuged at 6000×g for 10 min at 4°C. The pellets were washed twice in 200 μl of ice-cold PBS and resuspended in 200 μl of PBS. Then, 2 mM disuccinimidyl suberate (DSS) was added to the resuspended pellets, which were incubated at room temperature for 30 min with rotation. Samples were then centrifuged at 6000×g for 10 min at 4°C. The cross-linked pellets were resuspended in 40 μl of 2× loading buffer and then boiled and analyzed by immunoblotting.

**2.18.2 Immunoblot analysis**

Cells were lysed in NP-40 buffer containing a protease inhibitor cocktail (Roche, 11836153001) and a phosphatase inhibitor cocktail (Sigma-Aldrich, P5726). Protein concentrations were quantified using a BCA Protein Assay Kit (Beyotime, 5000T P0011). Equal amounts of protein were separated by SDS-PAGE and transferred onto nitrocellulose membranes. The membranes were then incubated with antibodies against CMPK2, NLRP3, ASC, pro-caspase-1, pro-IL-1β, GAPDH, caspase-1 and IL-1β, followed by incubation with the appropriate secondary HRP-conjugated antibodies and development with ECL.

**2.19 siRNA-mediated gene silencing and dsDNA-mediated gene overexpression in BMDMs**

BMDMs were plated in 6-well plates at a density of 1.5×10^6^ cells per well and then transfected with 150 nM siRNA using Lipofectamine RNAiMAX according to the manufacturer’s guidelines (Invitrogen). siRNA sequences were chemically synthesized by GenePharma, and the negative control siRNA was also from GenePharma. The siRNA sequences were as follows: sicmpk2 5’-CCAGGUCAUUGCCAUUGAATT-3’.

Full-length human CMPK2 was cloned and inserted into a pcDNA3.1(+) vector (Invitrogen) for expression in mouse BMDMs. An HA tag was placed after the C-terminal of CMPK2. Mouse BMDMs were transfected using Fugene HD Transfection Reagent (Promega) with the CMPK2 plasmid. BMDMs were plated in 6-well plates at a density of 1.5×10^6^ cells per well and then transfected with 6 μg dsDNA using FuGENE according to the manufacturer’s guidelines (Promega).

- 1. **LPS-induced septic shock model**

Sepsis was induced by intraperitoneal injection of 8- to 12-week-old gender-matched wild-type mice with LPS (*E. coli O111:B4*, Sigma-Aldrich) at 20 mg per kg body weight. Mouse survival was monitored every 1 h after injection for a total of 72 h. In a separate experiment, mice were treated with the same dose of LPS, and immune sera were collected 6 h post injection. Then, the abdominal cavity was washed with PBS. The levels of IL-1β and TNF-α in serum and peritoneal lavage ﬂuid were determined by ELISA. Then, the livers, kidneys and lungs were immediately removed and washed in ice-cold PBS. They were fixed with 4% paraformaldehyde, which was used for hematoxylin-eosin (H&E), Masson staining and Sirius red staining.

- 1. **Chemometric data and statistical analysis**

**2.23.1 Chemometric data analysis**

The ESI^−^ and ESI^+^ raw data were analyzed by PeakView Applications Manager version 2.2 (SCIEX, USA). The multivariate data matrix was imported into SIMCA-P 14.1 software for unsupervised principal component analysis (PCA). MetaboAnalyst 5.0 was used with the general line model procedure to evaluate significant differences between the ligand-to-BSA and ligand-to-CMPK2 data. The level of statistical significance was set as *p* < 0.05*.* These results were analyzed using GraphPad Prism (ver. 5.0; GraphPad Software, Inc., San Diego, CA, USA).

The collected MS datasets were processed using MaxQuant (version 1.6.7) with a database containing the full-length sequences of CMPK2. The quantification multiplicity was set as 3, and the doublet quantification mode was selected, with dimethyl Lys 0 as the light label and dimethyl Lys 4 (modified to +32.0535 Da) as the heavy label. Chymotrypsin was set as the enzyme, with up to four missed cleavages allowed. The function of the match between runs was enabled. Methionine oxidation and acetylation of the protein N-terminus were set as variable modifications. The precursor ion mass tolerance was set at 10 ppm, and the fragment ion mass tolerance was 0.5 Da. The false discovery rate (FDR) of both peptide and protein identification was below 1%. The other parameters were set as default values.

- - 1. **Quantification and statistical analysis**

GraphPad Prism 7.0 and 9.0 were used to collect and analyze the data. Statistical analysis was performed with an unpaired *t* test when comparing two different groups or one-way ANOVA with Tukey’s multiple comparison tests. All calculated values are shown as the mean ± standard deviation (SD).


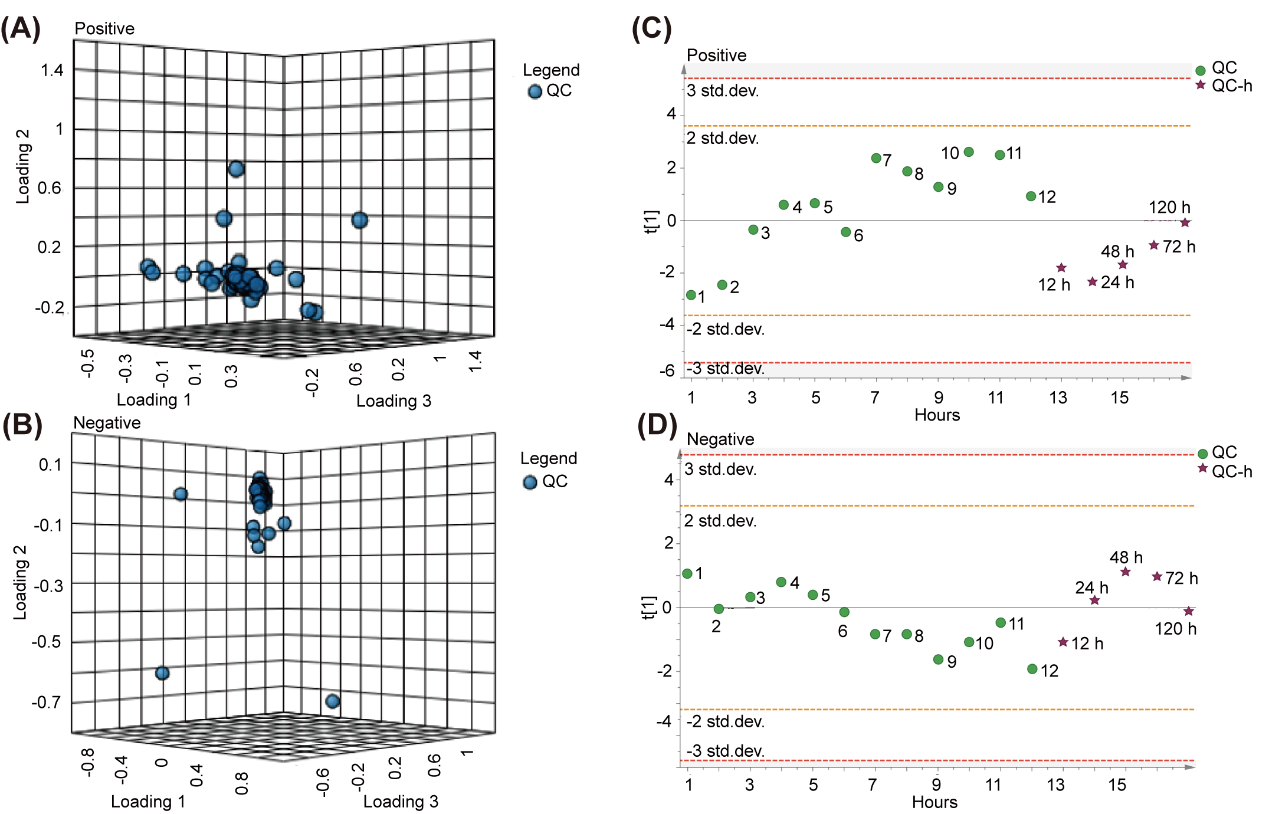


**Figure S1 PCA score plot of QC samples.** (A) The aggregation trend graph of QC samples in positive ion detection mode. (B) The aggregation trend graph of QC samples in negative ion detection mode. (C and D) PCA score plots of QC samples for method validation in positive and negative ion detection modes. QC-h: QC samples were placed within 120 h at 4℃.


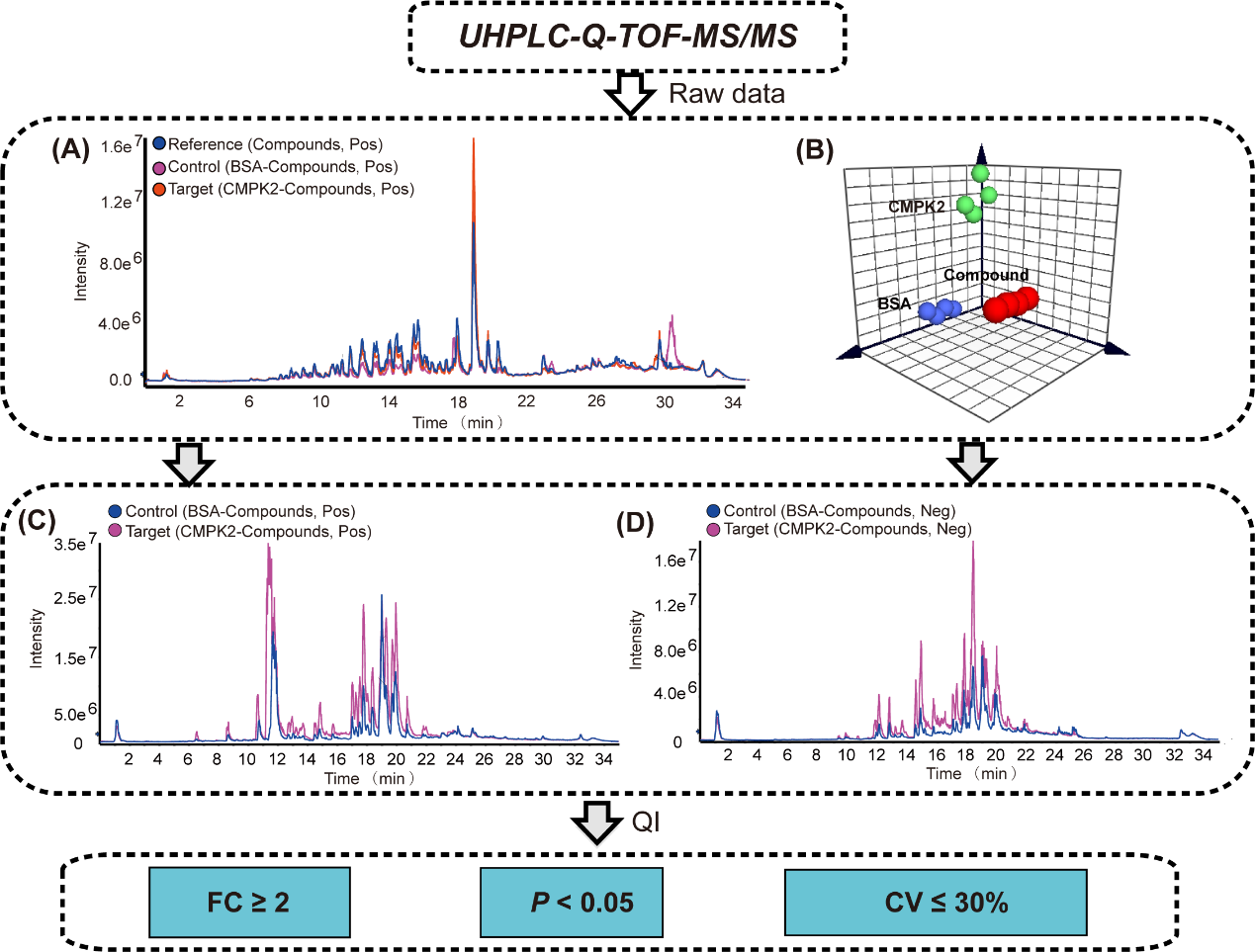


**Figure S2 Prejudgment flowchart of LC-MS data processing and chemometrics statistical analysis.** (A) Representative LC-MS chromatograms of cocktails, CMPK2 target, and control. (B) Multivariate analysis of the LC-MS data with PCA mode. (C and D) Representative LC-MS chromatograms in positive and negative ion detection modes.


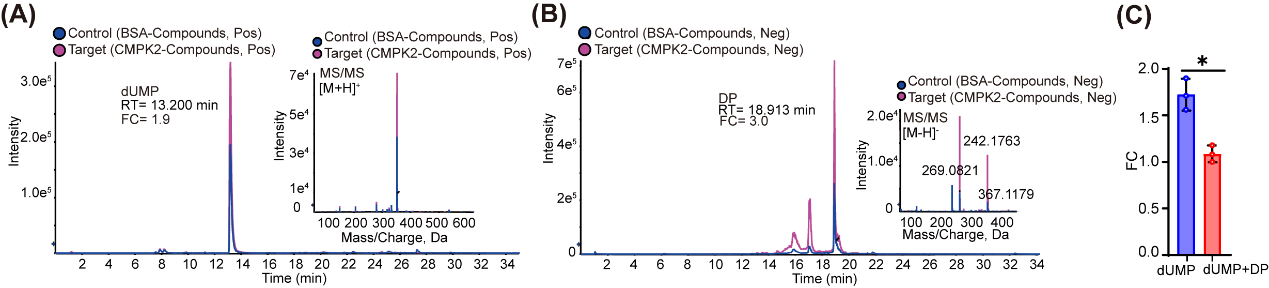


**Figure S3 FC derived from LC-MS measurements and typical MS/MS spectra of the dUMP (A) and DP (B).** (C) DP and DUMP jointly compete for the pocket of CMPK2 kinase. Statistics were analyzed using an unpaired Student’s *t* test: *, *P* < 0.05; **, *P* < 0.01; ***, *P* < 0.001. NS, not significant.


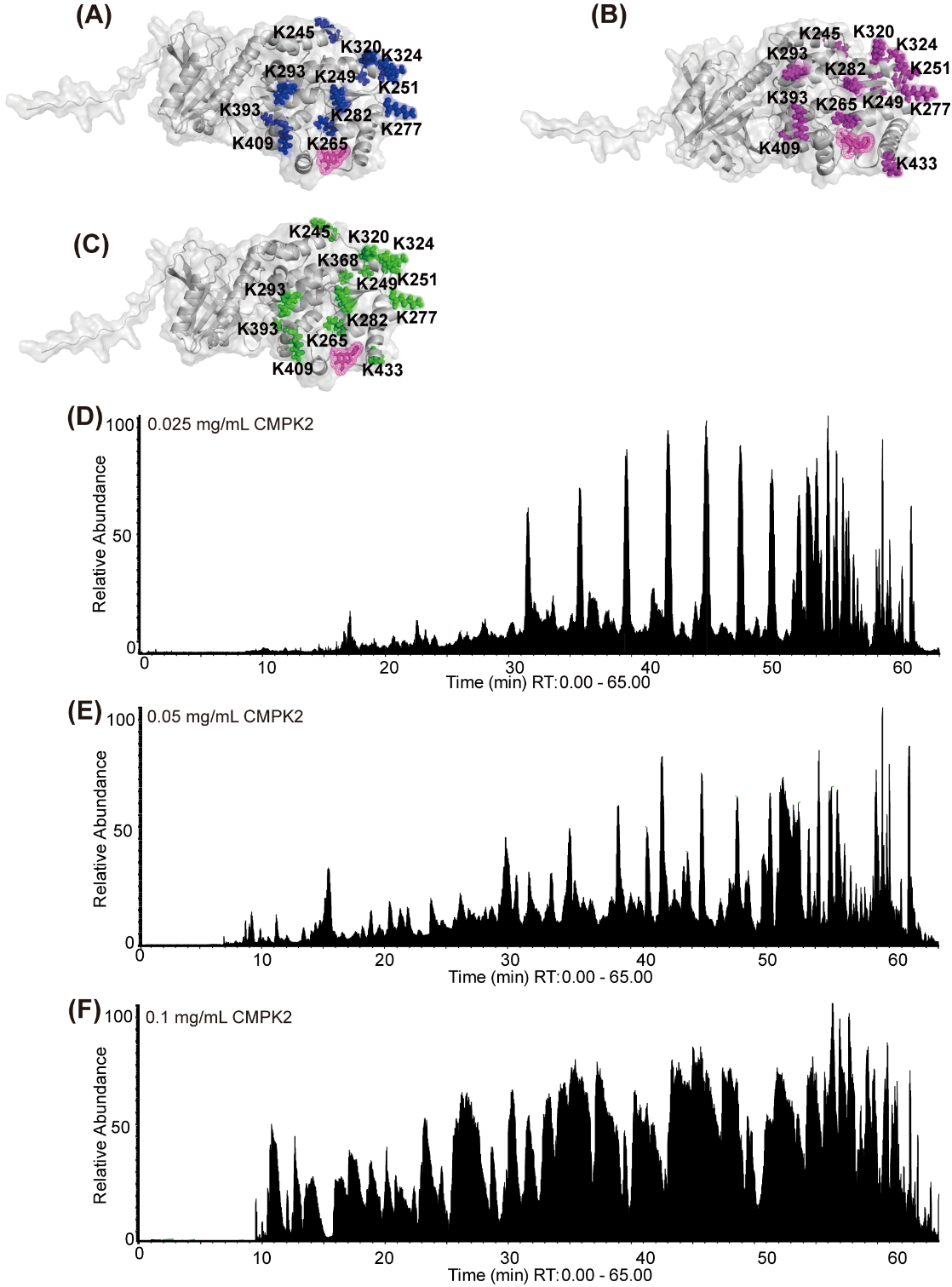


**Figure S4 Total extraction ion chromatograms of different concentrations of CMPK2 kinase complex.** (A, B and C) Dimethyl labeling time was 5, 10 and 15 min, the conformational distribution of the quantified lysine sites. (D) Total extracted ion chromatograms of 0.025 mg/ml CMPK2 kinase complex. (E) Total extracted ion chromatograms of 0.05 mg/ml CMPK2 kinase complex. (F) Total extracted ion chromatograms of 0.1 mg/ml CMPK2 kinase complex.


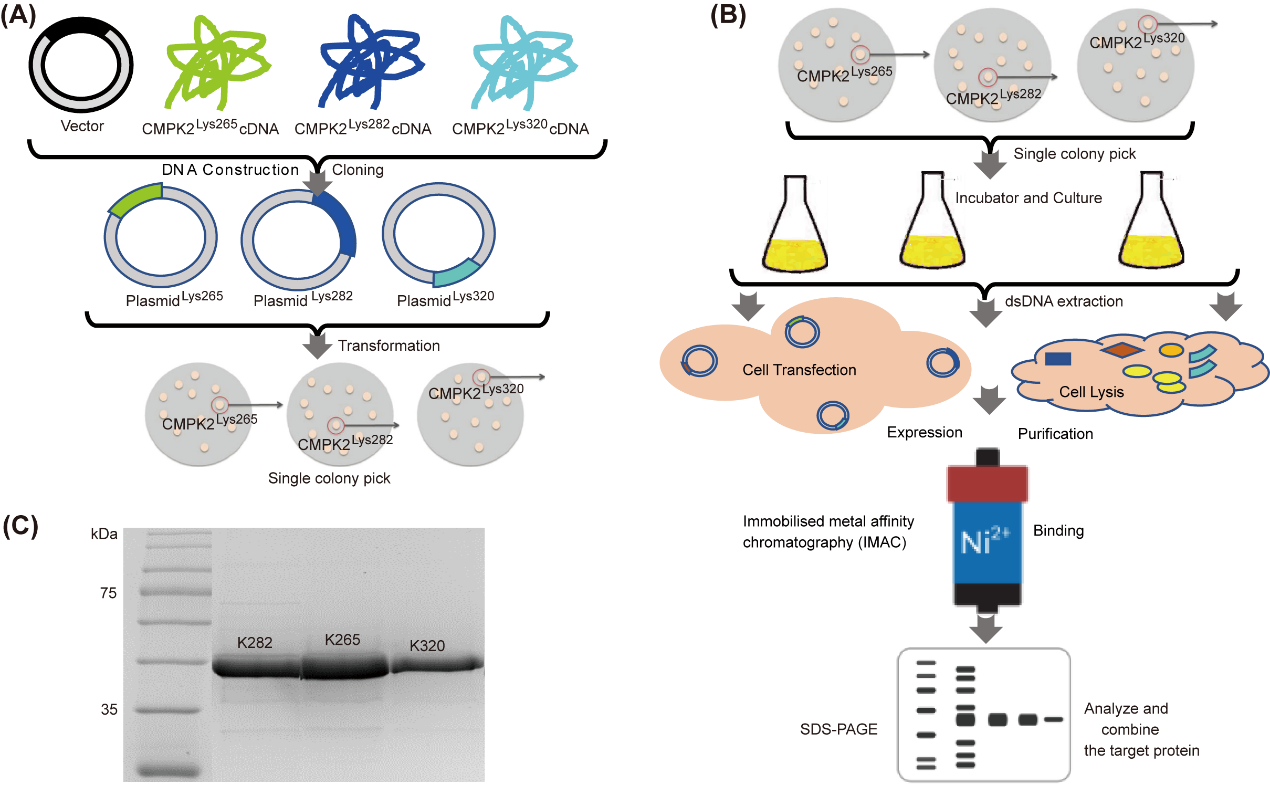


**Figure S5 Expression and purification of recombinant CMPK2^Lys265^, CMPK2^Lys282^ and CMPK2^Lys320^ kinase.** (A) Construction of CMPK2^Lys265^, CMPK2^Lys282^ and CMPK2^Lys320^ mutant kinase plasmids. (B) Expression and purification flow chart of CMPK2^Lys265^, CMPK2^Lys282^ and CMPK2^Lys320^ mutants. (C) The expression of CMPK2^Lys265^, CMPK2^Lys282^ and CMPK2^Lys320^ mutants was visualized by SDS-PAGE.


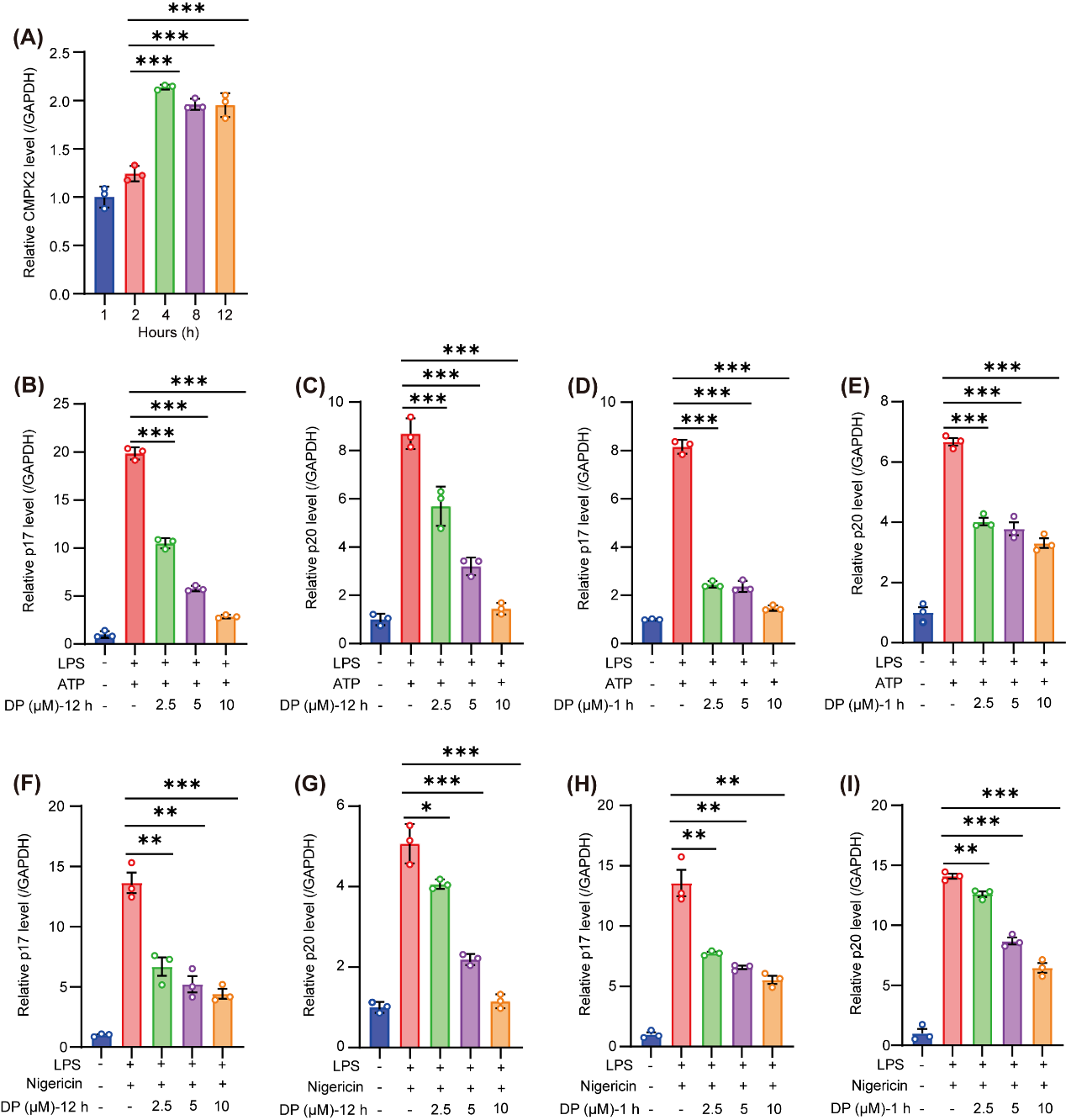


**Figure S6 Statistical assay of the relative protein contents in FIGURE 5.** (A) Time-course analysis of CMPK2 accumulation in wild-type BMDMs after LPS priming. (B, C, F and G) DP inhibited the expression of CMPK2 in BMDMs. BMDMs were treated with various doses (above lanes) of DP (2.5, 5 and 10 μM) for 12 h, and then LPS-primed BMDMs were stimulated with 4 mM ATP or 10 μM Nigericin, corresponding to the statistical blots for p17 and p20. (D, E, H and I) LPS-primed BMDMs treated with various doses of DP for 1 h and then stimulated with 4 mM ATP or 10 μM Nigericin, corresponding statistical graphs of p17 and p20 blots. Statistics were analyzed using an unpaired Student’s *t* test: *, *P* < 0.05; **, *P* < 0.01; ***, *P* < 0.001. NS, not significant.**
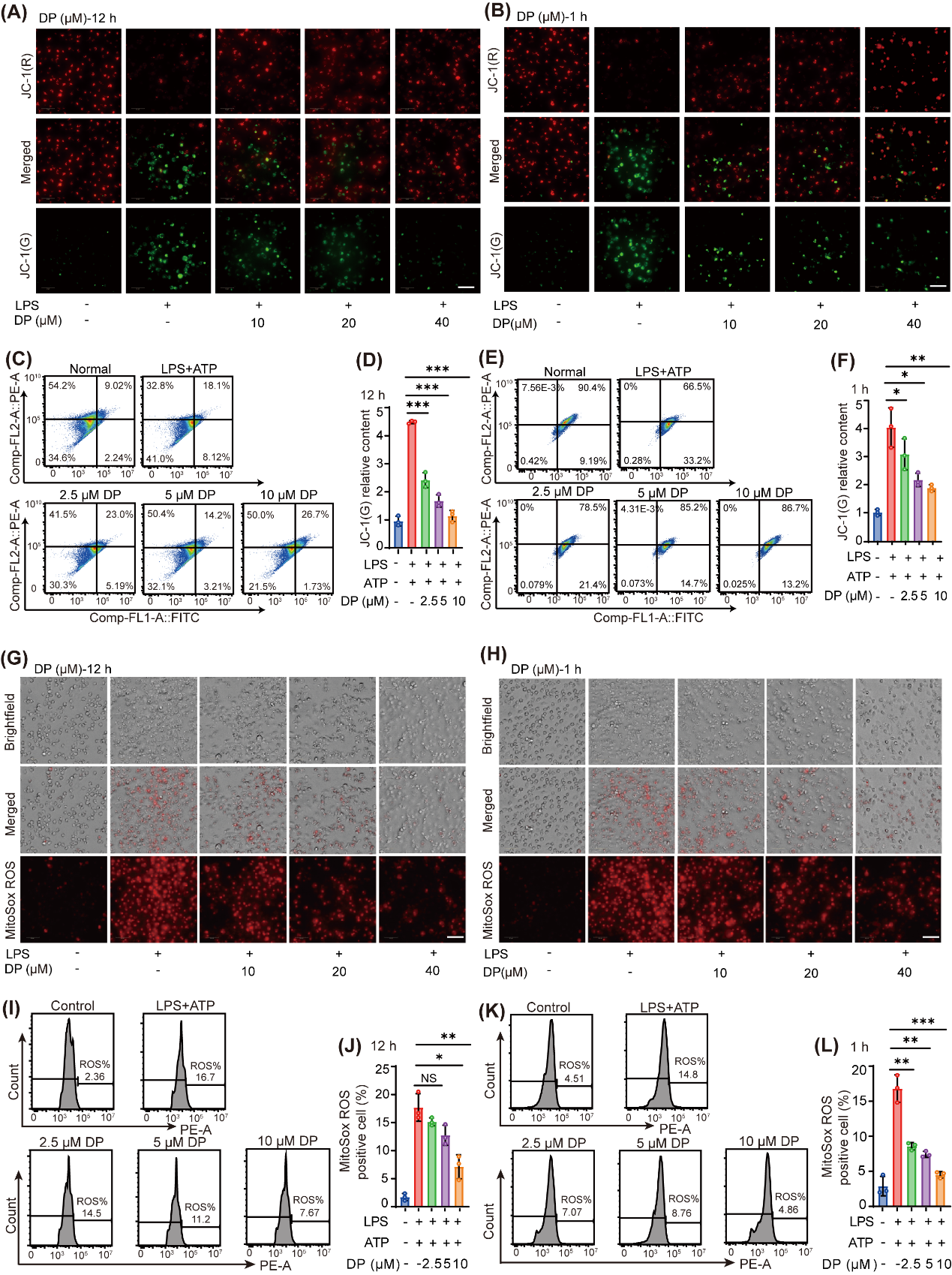
**

**Figure S7 DP blocked the LPS/ATP-induced decrease in mitochondrial damage.** BMDMs were treated with different doses of DP for 12 h and then stimulated with LPS for 4 h (DP before LPS), or BMDMs were treated with LPS for 4 h and stimulated with different doses of DP for 1 h (DP after LPS). (A and B) DP blocked the LPS/ATP-induced decrease in membrane potential (MMP), which was detected by JC-1 staining (scale bar: 50 μm). (C-F) DP blocked the LPS/ATP-induced decrease in MMP, which was detected by flow cytometry. (G and H) DP blocked the LPS/ATP-induced decrease in mitochondrial reactive oxygen species (mtROS), which was detected by the MitoSOX™ Red mitochondrial superoxide indicator (scale bar: 100 μm). (I-L) DP blocked the LPS/ATP-induced decrease in mtROS levels, which was detected by flow cytometry.

**
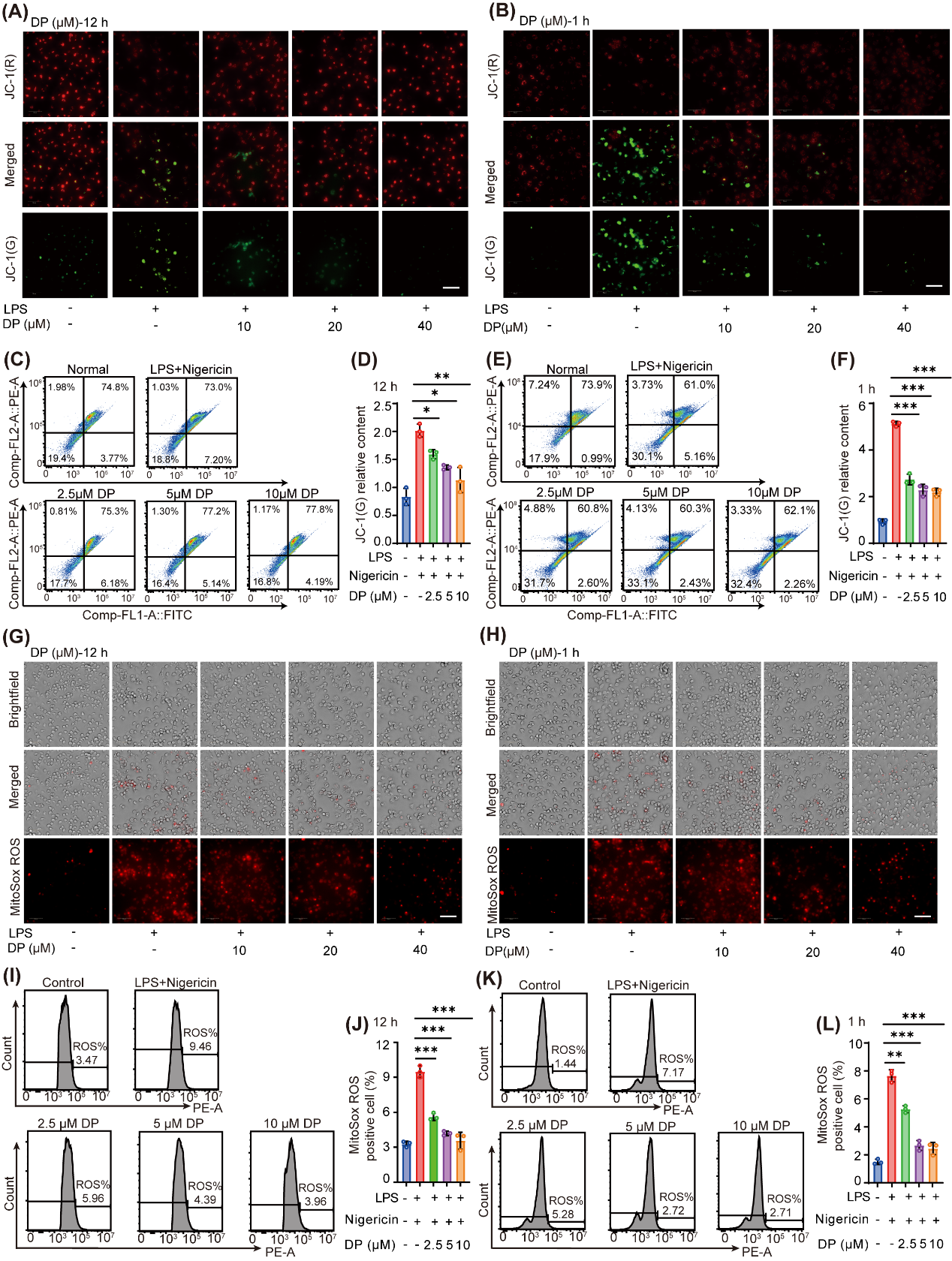
**

**Figure S8 DP blocked the LPS/Nigericin-induced decrease in mitochondrial damage.** BMDMs were treated with different doses of DP for 12 h and then stimulated with LPS for 4 h (DP before LPS), or BMDMs were treated with LPS for 4 h and stimulated with different doses of DP for 1 h (DP after LPS). (A and B) DP blocked the LPS/nigericin-induced decrease in membrane potential (MMP), which was detected by JC-1 staining (scale bar: 50 μm). (C-F) DP blocked LPS/Nigericin-induced decrease in MMP, which was detected by flow cytometry. (G and H) DP blocked LPS/Nigericin-induced decrease in mitochondrial reactive oxygen species (mtROS), which was detected by the MitoSOX™ Red mitochondrial superoxide indicator (scale bar: 100 μm). (I-L) DP blocked LPS/Nigericin-induced decrease in mtROS, which was detected by flow cytometry.


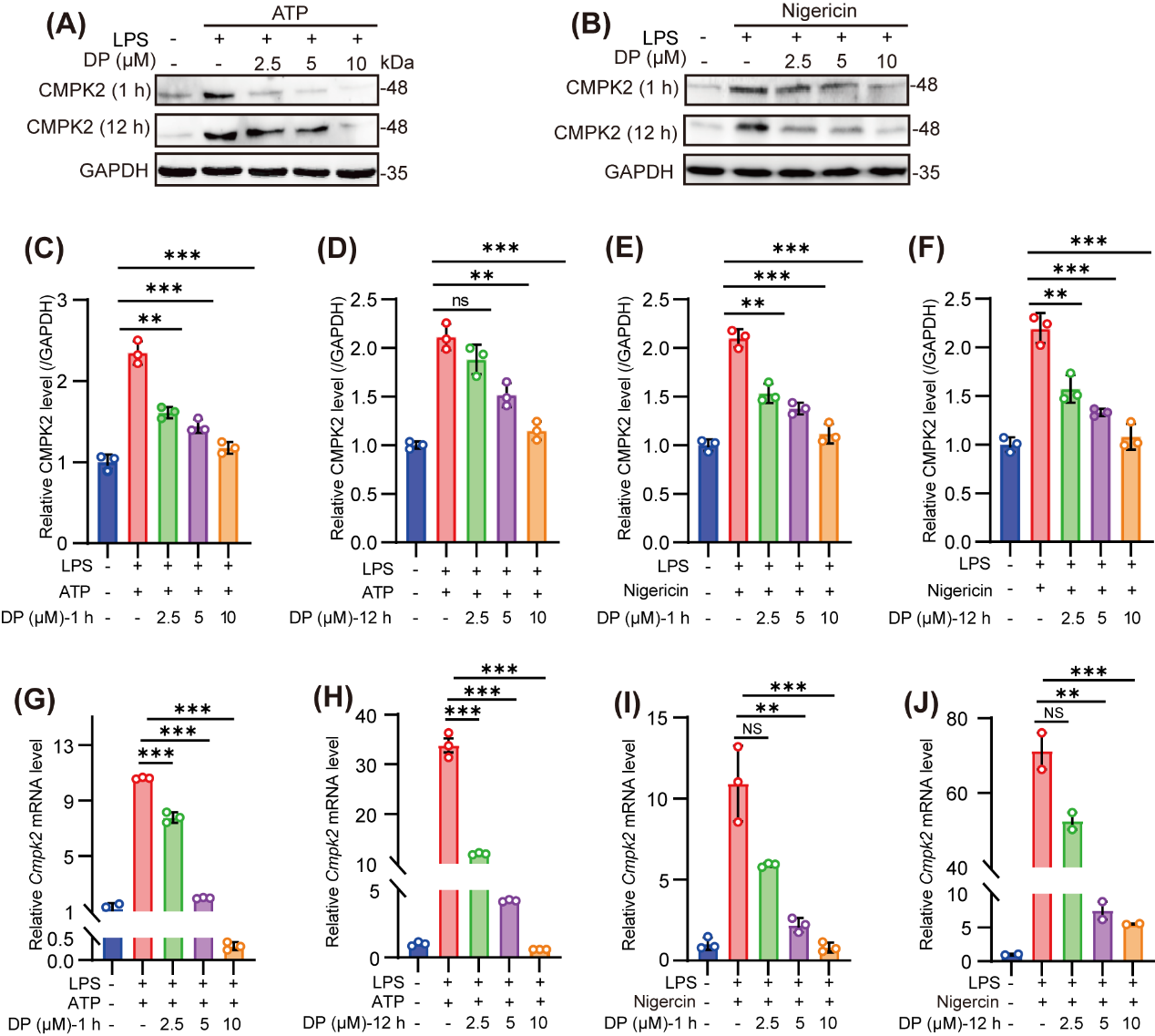


**Figure S9 DP inhibited the expression of CMPK2 in BMDMs.** (A and B) DP inhibited the expression of CMPK2 in BMDMs. BMDMs were treated with various doses (upper lanes) of DP (2.5, 5 and 10 μM) for 12 h, and then LPS-primed BMDMs were stimulated with 4 mM ATP or 10 μM Nigericin. LPS-primed BMDMs were treated with various doses of DP for 1 h and then stimulated with 4 mM ATP or 10 μM Nigericin, corresponding to blots of CMPK2. (C-F) Corresponding statistical blots for CMPK2. (G-J) DP decreased CMPK2 expression at the mRNA level. Statistics were analyzed using an unpaired Student’s *t* test: *, *P* < 0.05; **, *P* < 0.01; ***, *P* < 0.001. NS, no significance.


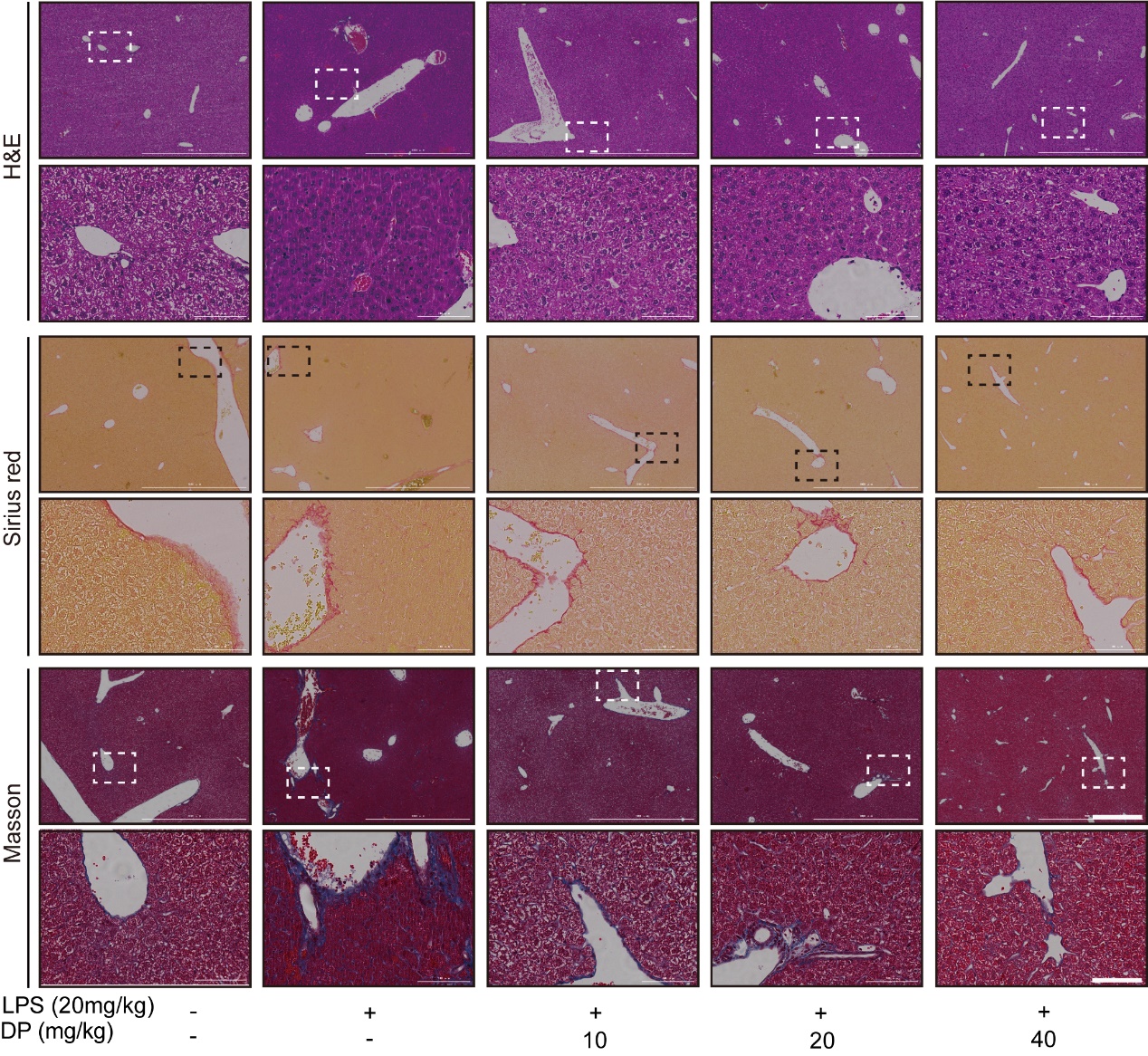


**Figure S10 H&E staining, Sirius red staining and Masson staining of liver tissue from mice that were left untreated or pretreated with 10, 20 and 40 mg/kg DP and challenged with 20 mg/kg LPS 24 h prior to tissue collection. Scale bar, 100 μm and 20 μm. n=10 mice per group.**


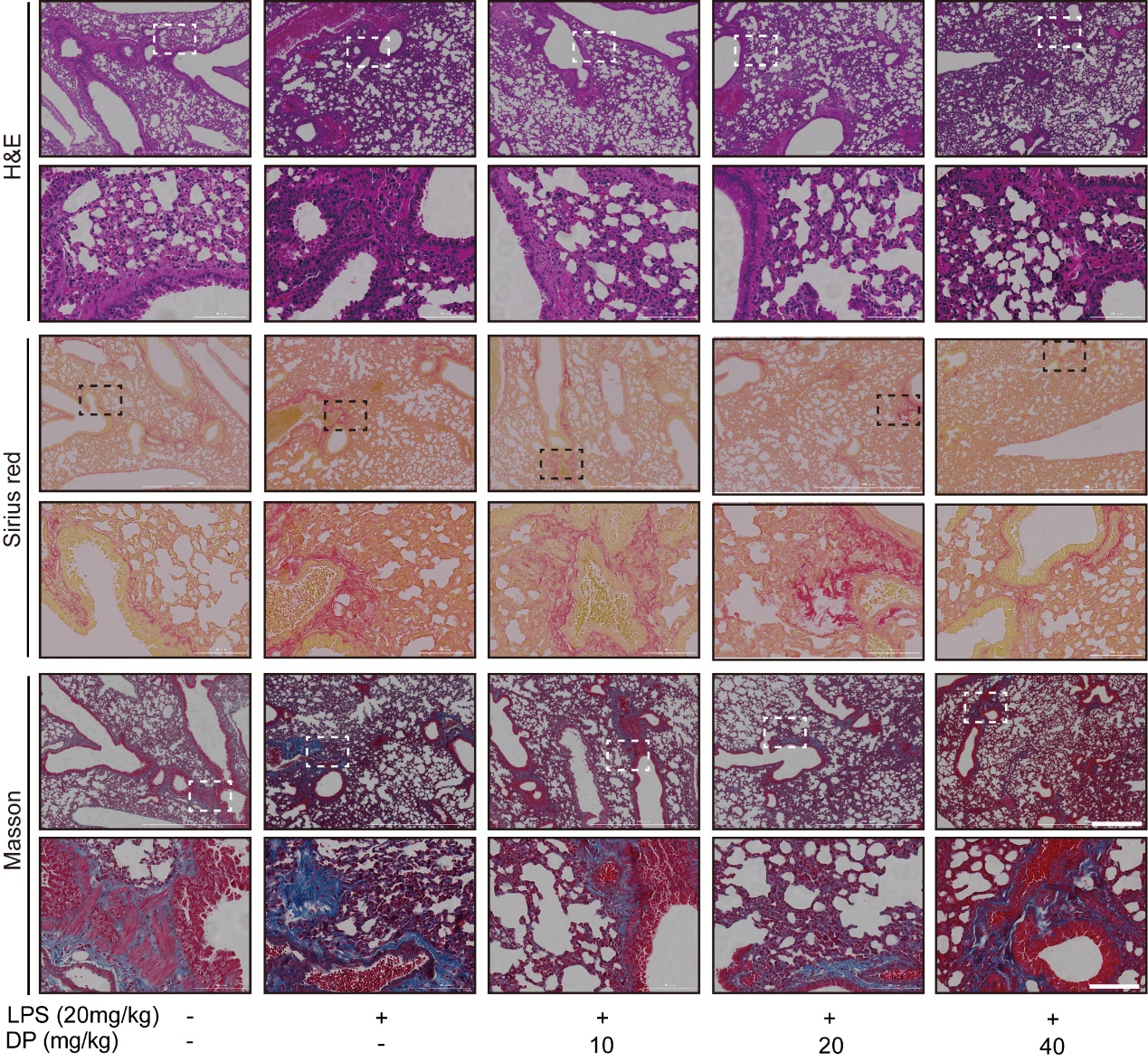


**Figure S11 H&E staining, Sirius red staining and Masson staining of lung tissue from mice that were left untreated or pretreated with 10, 20 and 40 mg/kg DP and challenged with 20 mg/kg LPS 24 h prior to tissue collection. Scale bar, 100 μm and 20 μm. n=10 mice per group.**


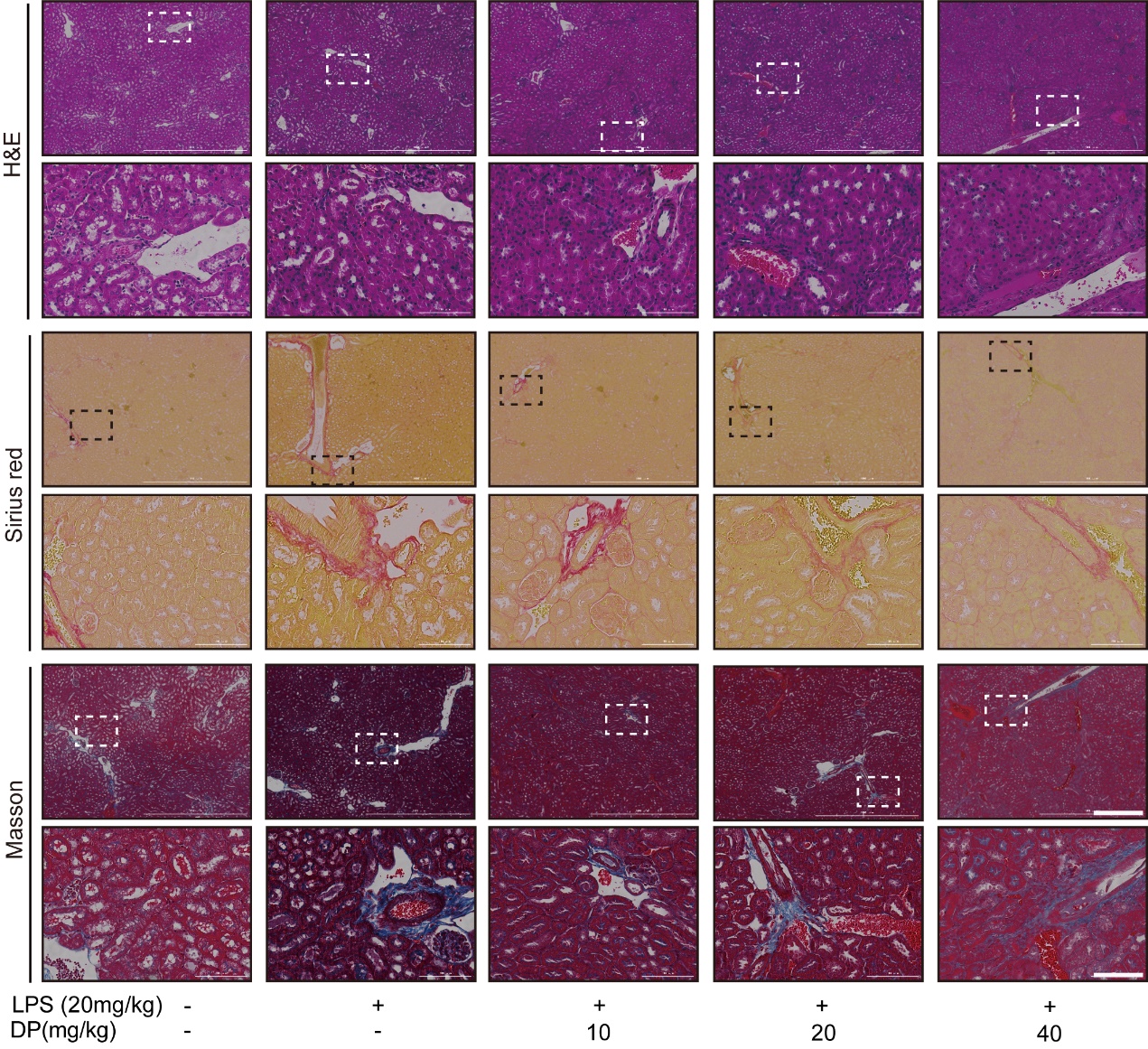


**Figure S12 H&E staining, Sirius red staining and Masson staining of** **kindey tissue from mice that were left untreated or pretreated with 10, 20 and 40 mg/kg DP and challenged with 20 mg/kg LPS 24 h prior to tissue collection. Scale bar, 100 μm and 20 μm. n=10 mice per group.**

**
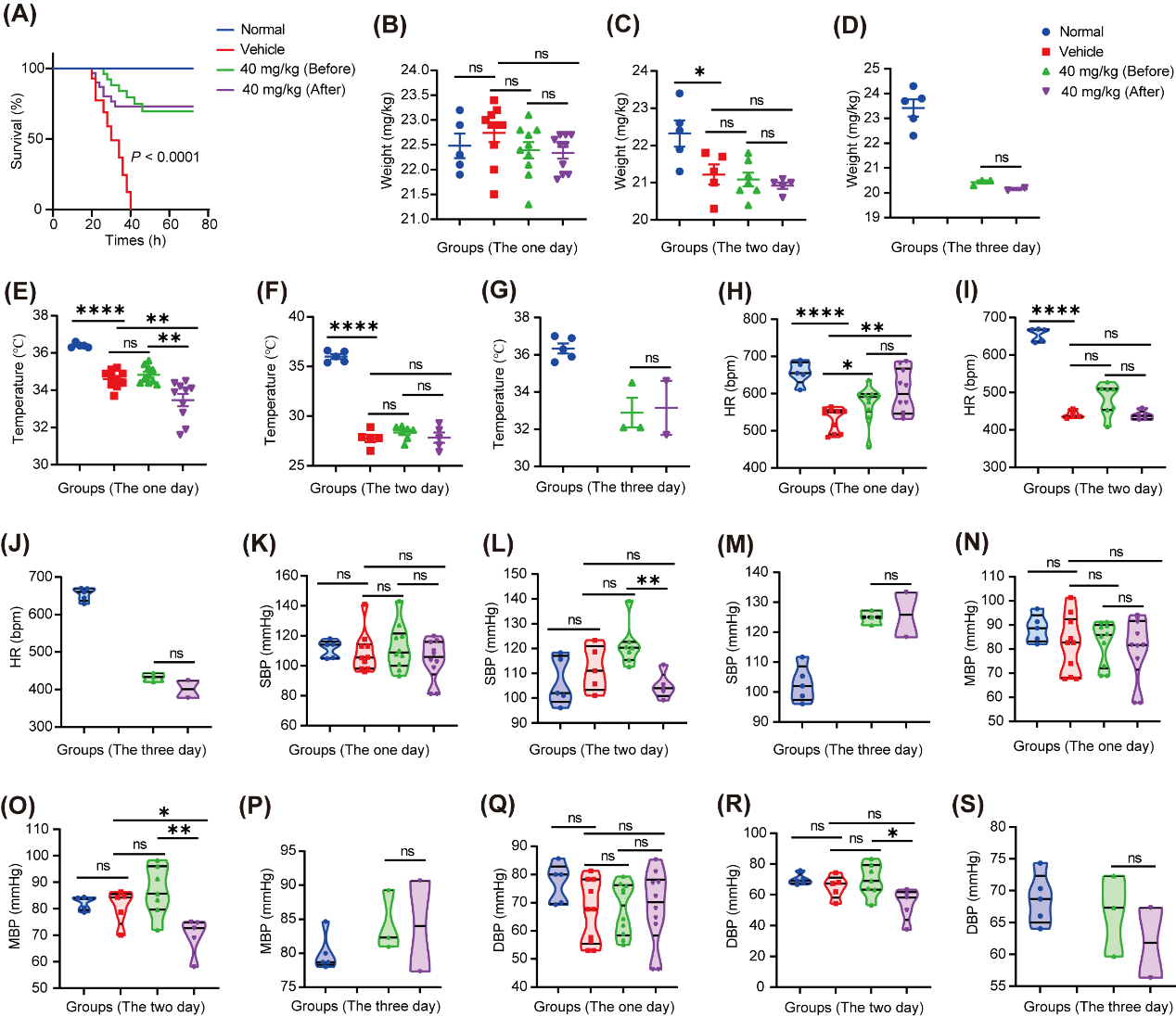
Figure S13 Changes in phenotypic indexes, such as the survival curve, blood pressure, heart rate, body temperature and body weight, in mice after LPS injection.** (A) Survival curve within 72 hours. Red line: Eight-week-old C57BL/6 male mice were treated with DP (40 mg/kg), and two hours later, they were i.p. injected with LPS (20 mg/kg). Blue line: Eight-week-old C57BL/6 male mice were treated with vehicle, and two hours later, they were i.p. injected with LPS (20 mg/kg). Green line: Eight-week-old C57BL/6 male mice were treated with LPS (20 mg/kg), and one hour later, they were i.p. injected with DP (40 mg/kg). The survival of the mice was monitored for 72 h (n = 10/group). (B-D) Body weight within 72 hours. (E-G) Body temperature within 72 hours. (H-J) Heart rate within 72 hours. (K-M) Systolic blood pressure (SBP) within 72 hours. (N-P) Mean blood pressure (MBP) within 72 hours. (Q-S) Diastolic blood pressure (DBP) within 72 hours.


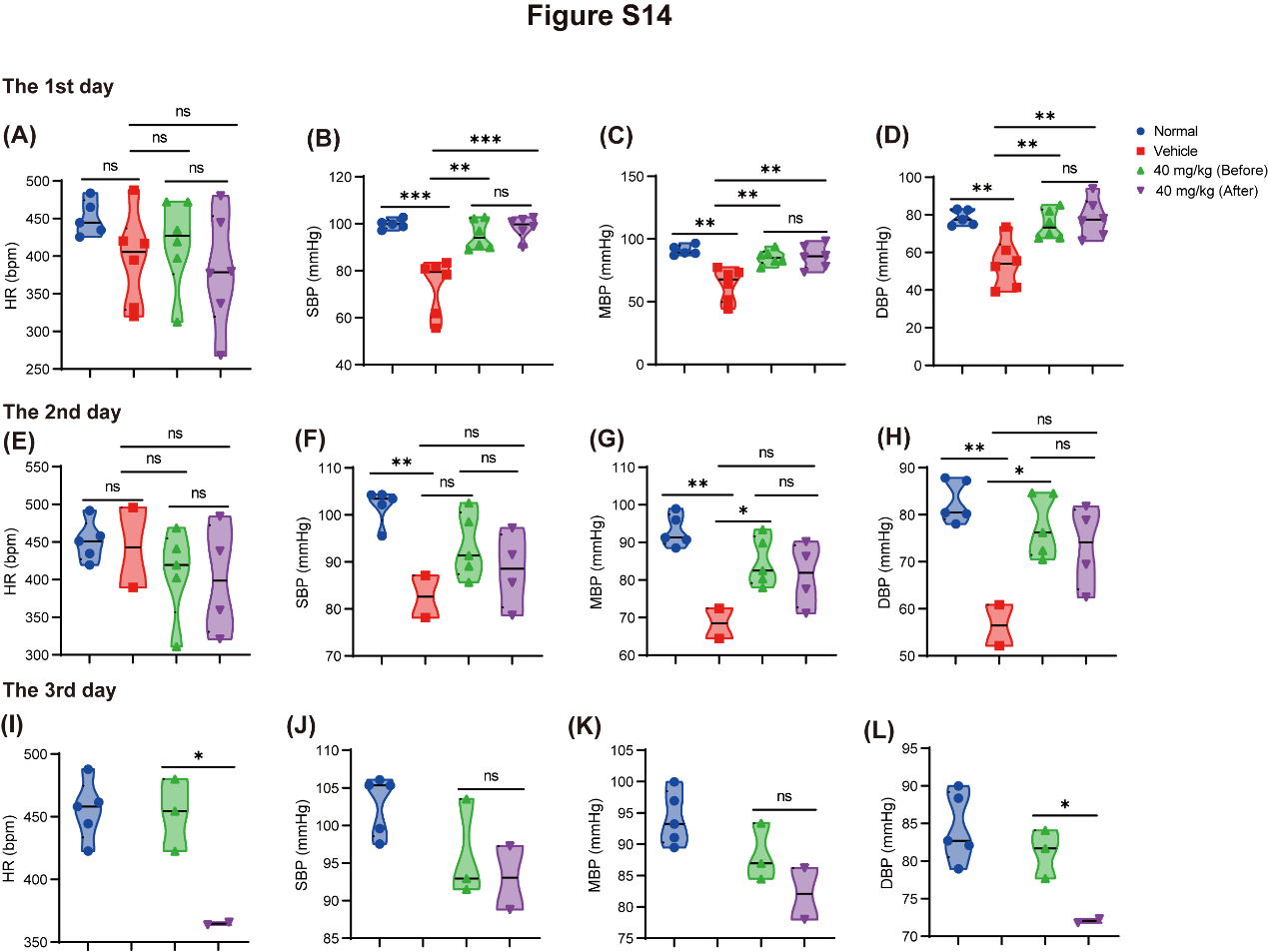


**Figure S14 Changes in phenotypic indexes, such as blood pressure and heart rate, in mice after LPS injection.** (A, E and I) Heart rate within 72 hours. (B, F and J) Systolic blood pressure (SBP) within 72 hours. (C, G and K) Mean blood pressure (MBP) within 72 hours. (D, H and L) Diastolic blood pressure (DBP) within 72 hours.
