# Supplementary material for: Dracorhodin targeting CMPK2 attenuates inflammation: A novel approach to sepsis therapy
Source: Clin Transl Med. 2023 Oct 20;13(10):e1449. doi: 10.1002/ctm2.1449 (PMC10587737; doi:10.1002/ctm2.1449)
Supplement: Supplementary file 2 — Supporting Information [file CTM2-13-e1449-s001.docx]

**Supplementary Tables**

**Table S1 The precision of the QC samples**

| **Origin** | **Compounds** | **Positive RSD (%)** | | **Negative RSD (%)** | |
| --- | --- | --- | --- | --- | --- |
|  |  | **Retention times** | **Peak Area** | **Retention times** | **Peak Area** |
| CMPK2 | C-1 | 0.028 | 1.471 | 0.046 | 0.208 |
|  | C-2 | 2.134 | 3.231 | 0.731 | 0.601 |
|  | C-3 | 0.059 | 1.644 | 0.151 | 0.138 |
|  | C-4 | 0.086 | 1.035 | 0.904 | 0.230 |
|  | C-5 | 0.008 | 0.974 | 0.167 | 0.089 |
| BSA | C-1 | 0.045 | 2.055 | 0.032 | 0.874 |
|  | C-2 | 0.717 | 3.193 | 0.792 | 0.410 |
|  | C-3 | 0.046 | 1.810 | 0.077 | 1.290 |
|  | C-4 | 0.024 | 2.920 | 0.791 | 0.665 |
|  | C-5 | _ | _ | 0.031 | 1.231 |
| Ligand | C-1 | 0.031 | 0.319 | _ | 1.159 |
|  | C-2 | 0.990 | 1.980 | _ | 3.235 |
|  | C-3 | _ | _ | _ | _ |
|  | C-4 | 0.398 | 0.463 | 0.928 | 0.456 |
|  | C-5 | _ | 0.049 | 0.046 | 1.502 |

The *m/z* values of the compounds in the samples were C-1: 424.7000, C-2: 176.1700, C-3: 426.7174, C-4: 304.2500, and C-5: 458.5440. _: Not detected

**Table S2** **Detailed information about these four ligands of CMPK2**

| **Number** | **Name** | **Molecular formula** | **Molecular weight** | **FC** | | ***P*** | | ***RSD*** | |
| --- | --- | --- | --- | --- | --- | --- | --- | --- | --- |
|  |  |  |  | **Positive** | **Negative** | **Positive** | **Negative** | **Positive** | **Negative** |
|  | dUMP | C_9_H_11_N_2_Na_2_O_8_P | 352.15 | 1.17 | 1. 91 | 0.001 | 0.022 | 7.31 | 7.39 |
| 73 | Dracohodin perochlorate | C_17_H_15_ClO_7_ | 366.75 | 4.32 | 3.00 | 0.003 | 0.015 | 5.79 | 3.31 |
| 368 | Protopanaxadiol | C_30_H_52_O_3_ | 460.74 | - | 3.70 | 0.013 | 0.029 | 7.01 | 5.90 |
| 400 | Hyperoside | C_21_H_20_O_12_ | 464.38 | 4.74 | 6.41 | 0.028 | 0.009 | 2.26 | 8.79 |
| 792 | 7-methoxy coumarin | C_10_H_8_O_3_ | 176.17 | 3.13 | - | 0.008 | 0.025 | 9.20 | 4.53 |

**Table S3 Predicted key interacting residues and affinity ΔG**

| **Number** | **Name** | **affinity (kcal/mol)** | **Amino acid** |
| --- | --- | --- | --- |
|  | dUMP | -7.0 | THR-266, GLY-264, LYS-265, THR-263, LYS-282 |
| 73 | Dracohodin perochlorate | -8.2 | TRP-291, LYS-282, LYS-265 |
| 368 | Protopanaxadiol | -6.6 | GLU-399 |
| 400 | Hyperoside | -7.0 | GLN-387, ASP-261, ARG-389, ARG-331, LYS-265 |
| 792 | 7-methoxy coumarin | -7.4 | ARG-304 |

**Table S4 Identified K-containing peptides after incubation with different concentrations of CMPK2**

| **CMPK2 (mg/ml)** | **Sequence** |
| --- | --- |
| 0.025 | / |
| 0.05 | SLGNYIVASEIAKESAKSPVIVDRY |
|  | DLVDQCPKQIQKGKF |
|  | RQKVEMSY |
|  | RKIFDDEPTIIRRAFY |
|  | DATGKTTVTQSVADSLKAVLL |
|  | KSPPSCIGQWRKIFDDEPTIIRRAFY |
|  | QGRGMEKTREEAELEANSVF |
| 0.1 | SLGNYIVASEIAKESAKSPVIVDRY |
|  | DLVDQCPKQIQKGKF |
|  | RQKVEMSY |
|  | RKIFDDEPTIIRRAFY |
|  | DATGKTTVTQSVADSLKAVLL |
|  | KSPPSCIGQWRKIFDDEPTIIRRAFY |
|  | QGRGMEKTREEAELEANSVF |
|  | QVVAIEGLDATGKTTVTQSVADSL |
|  | QRMENPGCHVVDASPSREKVLQTVL |
|  | KSPPSCIGQW |
|  | KAVLLKSPPSCIGQW |
|  | RQKVEMSYQRMENPGCHVVDASPSREKVLQTVL |
